# Microbial Succession, Functional Dynamics, and Their Relationship with Quality Formation During Ripened Pu-Erh Tea Fermentation

**DOI:** 10.3390/foods15173162

**Published:** 2026-09-07

**Authors:** Xinya Chen, Tianyu Wu, Xinghua Wang, Xiujuan Deng, Junjie He, Yuqing Li, Kai Peng, Chunyan Zhao, Changmin Cai, Yimeng Zhang, Baijuan Wang, Raoqiong Che

**Affiliations:** 1 College of Tea Science, Yunnan Agricultural University, Kunming 650201, China; chenxinya128@163.com (X.C.); wty15508851042@163.com (T.W.);; 2 Yunnan Organic Tea Industry Intelligent Engineering Research Center, Kunming 650201, China; 3 College of Agriculture, Yunnan Agricultural University, Kunming 650201, China; 4 Yunnan Liuda Chashan Tea Industry Co., Ltd., Kunming 650224, China

**Keywords:** microbial community, tea quality, metabolite transformation, pile fermentation

## Abstract

Microorganisms are the core drivers of ripened Pu-erh tea (RpPT) quality formation. However, the mechanisms of the microbiological and flavor chemical changes during the fermentation process remain unclear, which limits the quality control of ripened Pu-erh tea fermentation. A physicochemical analysis revealed that fermentation significantly reduced the contents of water extracts, tea polyphenols, free amino acids, soluble sugars, and most catechins (*p* < 0.05), while flavonoids and tea pigments were significantly accumulated. Amplicon sequencing revealed that *Pantoea* and *Bacillus* were the dominant bacterial genera during the early and late fermentation stages, with maximum relative abundances of 97.89% and 63.66%, respectively. *Aspergillus* and *Thermomyces* were the dominant fungal genera, reaching maximum relative abundances of 98.34% and 62.59%, respectively. During fermentation, bacterial diversity initially increased and then declined, whereas fungal diversity dropped sharply and subsequently stabilized. Co-occurrence network analysis indicated that microbial interactions were primarily cooperative, with network complexity gradually diminishing as fermentation progressed. Functional prediction showed enhanced microbial metabolism of amino acids and carbohydrates during the mid-to-late stages. Crucially, the stage-specific microbial communities were significantly correlated with distinct flavor compounds, shaping the final aroma profile of ripened Pu-erh tea. These dynamics explain the variations in tea quality and provide critical insights for optimizing the fermentation process.

## 1. Introduction

Tea is one of the most widely consumed beverages worldwide and is highly appreciated for its unique sensory properties and health-promoting effects. It is generally made from the leaves of the Camellia sinensis plant through processing techniques such as curing, rolling, heaping, and drying [1]. Yunnan is the birthplace of tea plants in the world, and it has its own unique geographical indication product—Pu-erh tea. According to the differences in processing techniques and product quality, Pu-erh tea can be divided into two categories: raw and ripened Pu-erh tea (RpPT) [2]. RpPT is a product formed by pile fermentation of Yunnan big-leaf sun-dried green tea under the influence of water, temperature and microbial action [3]. Compared with raw Pu-erh tea, RpPT has significant differences in the composition of substances, especially polyphenols and amino acids. RpPT exhibits a reddish-brown appearance with a distinctive aged aroma. Its infusion is bright and concentrated, with a mellow and sweet flavor profile, while the infused leaves remain uniformly reddish-brown. These sensory attributes collectively contribute to its favorable acceptance among consumers [4,5,6].

According to the production process and fermentation degree, RpPT belongs to post-fermented tea (dark tea). The unique “post-fermentation” technology not only results in the complex and abundant components of RpPT but also produces its unique flavor and taste, which affects the quality of RpPT. In fact, this fermentation technology influences the quality of RpPT carried out by microorganisms and their metabolic activities in several ways [7]. Microorganisms play an important role in the fermentation of tea, driving a series of reactions that modify the chemical constituents and thereby affect the flavor and bioactivities of tea [3]. Some microorganisms can produce beneficial compounds during the fermentation of food products. For example, *Lactic acid bacteria* can produce exopolysaccharides (EPSs) [8], *Eurotium cristatum* can generate organic acids and aroma-active alcohols [9], and *Bacillus species* can secrete proteases [10]. However, certain microorganisms may also produce undesirable compounds during fermentation. For instance, *lactic acid bacteria* are generally considered to be the major producers of biogenic amines in fermented foods; when these biogenic amine compounds accumulate excessively during an uncontrolled fermentation process, adverse reactions can occur [11].

Therefore, exploring how microorganisms influence the quality of RpPT is indispensable for developing accurate projections of industrialization and intelligent production of RpPT in the future. While most studies have focused on the influence of a single or several microorganisms in tea fermentation, less attention has been paid to the succession of microbial community diversity, composition, structure and function during the fermentation process and their relationship with the quality of RpPT at the population level [12]. The contribution of individual species to ecosystem functioning depends on their interactions with other microorganisms, and ecosystem functioning is collectively driven by fungal–bacterial diversity and microbiome complexity. In each of these biomes, microbes usually form highly diverse and complex communities that collectively function as a microbiome [13].

Recent advances in metagenomic and multi-omics approaches have greatly improved our understanding of the microbial ecology of RpPT fermentation. Integrated metagenomic and metabolomic analyses have revealed that microbial succession is closely associated with polysaccharide degradation, polyphenol transformation, and flavor formation during fermentation. However, the dynamic succession of bacterial and fungal communities throughout the fermentation process and their relationships with quality characteristics under practical production conditions remain insufficiently understood. Therefore, further investigation of microbial succession together with quality changes is still warranted [14]. Previous studies have confirmed that fungi and bacteria are the main groups in tea fermentation, and most of them are uncultured microorganisms [15,16,17]. Microorganisms exhibit significant differences in their tolerance to nonbiological environments. The fermentation process of RpPT takes place under high-temperature conditions. These unique environments lead to microbial selection, thereby shaping the microbial community structure. It is expected that this will change the microbial community of tea plants during their initial colonization and lead to different succession patterns over time. When microbial community composition changes over time, antagonistic and synergistic interactions among species also change, thus affecting the function of the microbial community and its effect on tea quality. It is thus uncertain how changes in the diversity, composition and function of the microbial community affect the fermentation process of RpPT and the relationship and mechanism between the microbial community and the quality formation of RpPT.

At present, most microorganisms are difficult to cultivate during food fermentation, and the special environmental conditions during the fermentation process further increase the difficulty of pure microbial cultivation. Furthermore, the accumulation of specific microorganisms and fermentation products in fermented foods may further influence the composition and function of the entire microbial community during the fermentation process. For instance, during the fermentation process, lactic acid bacteria produce biogenic amine compounds such as histamine and other substances. If the quantity of these products accumulates excessively, it can cause adverse reactions in the human body [11]. Thus, revealing the structure and function of the microorganism community through metagenomics would be important to elucidate the fermentation mechanism and quality formation mechanism of RpPT. The metagenomics approach is not limited to studies of single strains or a few strains combined in the fermentation process, which is more in line with actual industrial production. In actual production, the fermentation process of RpPT is a coordinated and collaborative effort of multiple strains. Therefore, it is more scientific and practical to study the impact of microorganisms on RpPT quality from the perspective of the microbial community. At the same time, the identification of core/key microbial communities, as well as the composition of the community as it evolves throughout the fermentation process, not only reveals the role of microorganisms in RpPT fermentation but also provides guidance for the fermentation process. However, we still do not know the evolutionary process, composition structure, and function of the microbial community during the entire process of RpPT fermentation, nor the mechanism by which the entire microbial community influences the formation of RpPT quality. Therefore, we propose the following scientific hypothesis: the dynamic succession of the entire microbial community (including both fungal and bacterial populations) during the post-fermentation process of RpPT is the primary driver of chemical transformation and final quality formation, and this community-level effect cannot be fully explained by the action of any single species alone.

Therefore, the present study aimed to systematically investigate the dynamic succession of microbial communities (including both fungal and bacterial populations) and their functions throughout the fermentation process of RpPT and to elucidate the mechanism correlations between dynamic changes at the microbial community level and the formation of key quality attributes. To achieve this, we performed metagenomic sequencing on 75 samples collected from four independent fermentation lines at multiple time points and comprehensively analyzed the taxonomic composition, community structure, and functional gene profiles of the associated microbiomes. Through these analyses, we sought to characterize the temporal trajectory of community assembly, decipher interspecies interactions, and establish correlative and potentially causal relationships between specific microbial successional phases and measurable quality parameters of RpPT. This study provides new insights into microbial ecological succession and its contribution to quality formation during industrial RpPT fermentation, while also establishing a theoretical basis for process optimization and intelligent quality control in RpPT.

## 2. Materials and Methods

### 2.1. Sample Collection, Shipping, and Classification

A total of 75 samples were collected from four fermentation lines (independent experimental groups) at two locations over the period from November 2023 to January 2024: Liuda Tea Mountain Tea Factory Co., Ltd. (Kunming, China) in Menghai County, Xishuangbanna Prefecture, and the Tea College of Yunnan Agricultural University, Kunming, Yunnan Province. Sampling was performed at each fermentation turning time point using the five-point sampling method, with 100–500 g of sample collected per point and three biological replicates established for each sample. The name of each sample was recorded based on the fermentation process or address, followed by the number of overturns, such as DF_0, DF_1, DF_2, etc. The samples were then transported back to the experiment in a foam box with dry ice and stored at −80 °C for subsequent microbial community analysis.

The fermentation process was classified into five stages to compare microbial community differences: initial (s), early (e), middle (m), late (a), and final (t). All fermentations treated samples at 0 and at the end of fermentation as beginning and ending samples, respectively. Because the four fermentation systems differed in fermentation duration and operational management, the sampling stages were defined according to the characteristic fermentation process of each production line rather than absolute fermentation time. Specifically, samples were collected at five representative developmental stages corresponding to the initial, early, middle, late, and final phases of fermentation, as determined by the operational process (e.g., pile turning), temperature profile, and fermentation progress of each production system. Therefore, comparisons among fermentation systems were performed based on biologically comparable developmental stages rather than identical fermentation days. Due to the timing control of temperature and humidity, the fermentation time of digital fermentation (DF) is short, at only 28 days, and the first 7 d is regarded as the pre-fermentation stage, 14 d as the mid-fermentation stage, 21 d as the post-fermentation stage, and 28 d as the end of fermentation stage. However, due to the small amount of traditional small-batch fermentation (TF), the fermentation time is also shortened by 4 days compared with the mass fermentation period in the tea factory, and 35 days are shared. The samples from the first 10 d are regarded as the pre-fermentation stage, 14–20 d as the mid-fermentation stage, 27 d as the post-fermentation stage, and 35 d as the end of fermentation stage. The Menghai Factory of the Liuda Tea Mountain Tea Factory Co., Ltd. (SG) and the He Kai Manor (HK) adopt traditional fermentation with the same amount of time. All belong to a lot of fermentation, at a total of 39 days, and the samples from 7 days are regarded as the pre-fermentation stage, 14–21 days as the mid-fermentation stage, 27–35 as the post-fermentation stage, and 39 d as the end of fermentation stage.

### 2.2. Determination of Physicochemical Properties

The content of water extracts was determined in accordance with the National Standard of the People’s Republic of China (GB/T 8305) using an analytical balance (ME204, Mettler Toledo, Greifensee, Switzerland) and a constant-temperature drying oven (DHG-9070A, Shanghai Yiheng Scientific Instrument Co., Ltd., Shanghai, China) [14]. The total amount of polyphenols in the tea soup was determined by the folinphenol method in accordance with GB/T 8313 [18], with gallic acid (Sigma-Aldrich, St. Louis, MO, USA) as the standard and absorbance measured at 765 nm using a UV-Vis spectrophotometer (UV-2600, Shimadzu, Tokyo, Japan). Total free amino acids were determined according to National (GB/T 8314) [19], using the same spectrophotometer with the ninhydrin colorimetric method at 570 nm, and the standard curve was established with theanine (Sigma-Aldrich, USA). Total flavonoid was determined by the aluminum trichloride–nitrous acid–sodium method [20], with rutin (National Institutes for Food and Drug Control, Beijing, China) as the reference standard, and absorbance was recorded at 510 nm. Soluble sugar content was determined by the anthrone colorimetric method [21], using glucose (Sigma-Aldrich, USA) as the standard, with absorbance read at 620 nm. The components of catechins, caffeine, and flavonoids were all determined using high-performance liquid chromatography (HPLC) on an Agilent 1260 Infinity II system (Agilent Technologies, Santa Clara, CA, USA) equipped with a ZORBAX Eclipse Plus C18 column (4.6 × 250 mm, 5 μm) (Agilent Technologies, Santa Clara, CA, USA), following the method described by Zhao [22]. The mobile phase consisted of 0.1% formic acid in water and acetonitrile at a flow rate of 1.0 mL/min, with the column temperature maintained at 35 °C and the detection wavelength set at 280 nm. All standard curves were constructed using authentic standards (catechin, epicatechin, epigallocatechin gallate, caffeine, and flavonoid aglycones, all from Sigma-Aldrich, USA) at five concentration levels, with correlation coefficients (R^2^) > 0.999. Each sample was injected in triplicate, and the relative standard deviation (RSD) for repeated injections was maintained below 2%.

The contents of theaflavins, thearubigins and theabrownins were determined by systematic analysis [23]. Briefly, a 3 g tea sample was steeped in 125 mL of boiling water for 10 min, mixed, filtered, and cooled. For Solution A, 2 mL of extract was diluted to 25 mL with ethanol after adding 2 mL of saturated oxalic acid and 6 mL of water. For Solution B, 10 mL of extract was mixed with 10 mL of n-butanol, vortexed, and centrifuged, and 2 mL of the lower layer was similarly diluted to 25 mL. For Solution C, 10 mL of extract was mixed with 10 mL of ethyl acetate, vortexed, and centrifuged, and 5 mL of the upper layer was shaken with 5 mL of sodium bicarbonate and recentrifuged; 1.6 mL of the upper layer was diluted to 10 mL with ethanol. Using 95% ethanol as a blank, the absorbances of Solutions A, B, and C were measured at 380 nm with a spectrophotometer (UV-2600, Shimadzu, Japan).

### 2.3. Sensory Evaluation

#### 2.3.1. Traditional Sensory Evaluation

The sensory characteristics and quality of the RpPT samples were evaluated in accordance with the national standard Tea Sensory Evaluation Method (GB/T 23776-2018) [24]. The sensory evaluation panel consisted of five professional tasters; all members possessed at least five years of experience in tea sensory assessment and had obtained national professional taster certification. Prior to the formal evaluation, a two-day calibration training session was conducted, during which the tasters utilized representative mature Pu-erh tea samples from different fermentation stages to establish unified scoring criteria, thereby ensuring consistent interpretation of various sensory attributes. Each sample underwent three replicate evaluations on separate dates, and the intraclass correlation coefficient (ICC) was calculated to assess the repeatability among the tasters; only attributes with ICC values greater than 0.80 were retained for the final analysis. Briefly, 3 g of tea leaves was steeped in 150 mL of boiling water for precisely 5 min, followed by filtration to obtain a tea infusion. A panel of five trained sensory evaluation professionals conducted the analysis, evaluating key attributes including appearance (20%), liquid color (10%), aroma (25%), taste (35%), and infused leaves (10%), with the overall score calculated based on these weighted criteria. Each parameter was assessed using a 100-point scale, ensuring a comprehensive and standardized evaluation of the tea’s sensory profile [25].

#### 2.3.2. Electronic Nose

Prior to analysis, the E-nose system was preheated and calibrated using filtered air. For sample measurement, the headspace injection method was employed. Briefly, 3 g of a tea sample was mixed with 150 mL of boiling water in a 250 mL conical flask, which was then sealed and equilibrated for 30 min. The operational parameters were set as follows: purge time, 60 s; zero adjustment, 10 s; pre-sampling, 5 s; and data acquisition, 120 s (injection flow rate: 300 mL/min). Each sample was measured in triplicate, and the average sensor response value from 60 to 62 s was used for data analysis.

### 2.4. Volatile Compound Analysis

Volatile compounds were extracted by headspace solid-phase microextraction (HS-SPME) using a DVB/CAR/PDMS fiber (50/30 μm × 1 cm; Supelco, Bellefonte, PA, USA). An appropriate amount of each sample was placed in a 20 mL headspace vial with 4 mL of saturated NaCl solution and 10 μL of n-hexyl-d13 alcohol internal standard. Samples were incubated at 80 °C for 10 min and extracted at 80 °C for 25 min, followed by desorption at 250 °C for 5 min.

Volatile compounds were analyzed using an Agilent 8890A GC (Agilent Technologies, Santa Clara, CA, USA) coupled with a LECO Pegasus BT 4D GC × GC-TOF-MS system. A DB-Heavy Wax column (30 m × 250 μm × 0.5 μm) and an Rxi-5Sil MS column (2 m × 150 μm × 0.15 μm) were used as the first- and second-dimension columns, respectively. Helium was used as the carrier gas at 1.0 mL/min. The oven temperature was initially held at 50 °C for 2 min, increased to 230 °C at 5 °C/min, and held for 5 min. The secondary oven was maintained 5 °C above the primary oven, and the modulator was maintained 15 °C above the secondary oven with a modulation period of 6.0 s. The injector temperature was 250 °C.

For TOF-MS detection, the transfer line and ion source temperatures were both 250 °C. Electron ionization was performed at 70 eV with an acquisition rate of 200 spectra/s over an *m*/*z* range of 35–550. Volatile compounds were annotated using ChromaTOF v5.51 software based on mass spectral information and retention indices (RIs), with experimental RIs calculated using C7–C30 n-alkanes. The relative odor activity value (ROAV) was calculated based on the relative content and odor threshold of each compound, with the compound showing the highest ratio of relative content to odor threshold defined as ROAV = 100.

### 2.5. Microbial DNA Extraction and Amplicon Sequencing

We investigated the bacterial and fungal communities at different fermentation stages with MiSeq sequencing, targeting the 16S rRNA and ITS2 region amplicons, respectively. Genomic DNA was extracted by using a FastPure Soil DNA Isolation Kit (Omega Bio-Tek, Norcross, GA, USA) according to the instructions, and the extraction quality was evaluated by 1% (*m*/*v*) agarose gel electrophoresis for further PCR amplification. The 16S rRNA gene V3-V4 fragments were amplified using primer pairs 341F (5′-TCGTCGGCAGCGTCAGATGTGTATAAGAGACAG-3′) and 806R (5′-GTCTCGTGGGCTCGGAGATGTGTATAAGAGACAG-3′), while the fungal ITS2 region was amplified using ITS3 (5′-GCATCGATGAAGAACGCAGC-3′) and ITS4 (5′-TCCTCCGCTTATTGATATGC-3′) primer pairs. The PCR product was extracted from 2% agarose gel and purified using the PCR Clean-Up Kit (YuHua, Shanghai, China), according to the manufacturer’s instructions, and quantified using Qubit 4.0 (Thermo Fisher Scientific, Waltham, MA, USA). Purified amplicons were pooled in equimolar amounts and then paired-end sequenced on an Illumina PE300/PE250 platform (Illumina, San Diego, CA, USA) according to the standard protocols by Majorbio Bio-Pharm Technology Co., Ltd. (Shanghai, China).

### 2.6. Sequencing Assembly and Analysis

The bioinformatics analysis of the raw data includes splicing, quality control and optimization. Briefly, raw FASTQ files were de-multiplexed using an in-house perl script and then quality-filtered by fastp version 0.19.6 [25] and merged by FLASH version 1.2.7 [26]. To normalize sequencing depth across samples, all samples were rarefied to the minimum sequencing depth among the 75 samples, thereby eliminating bias arising from uneven sequencing depths in downstream alpha- and beta-diversity analyses. Although OTU-based methods have the drawback of lower resolution compared to ASV-based methods, considering comparisons with previous studies and usability, this study applied OTU-based methods in the microbial research of Pu’er tea. The optimized sequences were clustered into operational taxonomic units (OTUs) using UPARSE 7.1 with a 97% sequence similarity level. The most abundant sequence for each OTU was selected as a representative sequence [27]. Taxonomic classifications of obtained high-quality data from each sample were performed using Silv (Release 138, http://www.arb-silva.de, accessed on 3 December 2025). The functions of microorganisms were predicted using PICRUSt2 (V1.1.0, http://picrust.github.io/picrust/, accessed on 3 December 2025). All these analyses were completed on the Majorbio Cloud Platform (www.majorbio.com) using default parameters.

Principal coordinates analysis (PCoA) and hierarchical clustering based on the Bray–Curtis distance were performed to compare differences in the microbiota, and statistical significance was evaluated by adonis analysis (*p* < 0.05). Linear discriminant analysis effect size (LEfSe) (http://galaxy.biobakery.org/, accessed on 3 December 2025; LDA > 2.0, *p* < 0.05) was applied to identify the biomarker species in the different fermentation stages of RpPT.

The results are shown as mean ± standard deviation. The physicochemical properties, sensory data, E-nose data, and volatile metabolite datasets were statistically analyzed using one-way ANOVA. Data visualization was conducted using OriginLab 8.0 (Origin Corporation, Northampton, MA, USA) and GraphPad Prism 8.0 (GraphPad Software, Inc., La Jolla, CA, USA). The principal component analysis (PCA), partial least-squares discrimination analysis (PLS-DA), and two-way orthogonal partial least squares (O2PLS) analysis of VFCs were performed using SIMCA 14.0 software. Differential metabolites were identified based on the criteria of variable importance in projection (VIP > 1), fold change (FC ≥ 2 or ≤0.5), and statistical significance (*p* < 0.05). Correlation networks were constructed using the psych package, with the correlation threshold at |r| > 0.8 and *p* < 0.05 to generate the network via Gephi v0.8.2.

## 3. Results and Discussion

### 3.1. Changes in Physicochemical Properties During Fermentation of RpPT

Physicochemical properties play an important role in the flavor of RpPT, and they serve as direct indicators of the tea’s intrinsic quality [28]. Our results demonstrated that the physicochemical properties of tea showed significant changes during fermentation (Figure S1a,b; Table S1). The relative contents of water extracts, tea polyphenols, free amino acids, and soluble sugars showed significant decreases in all samples (Figure S1a; Table S1). Leachates determine the thickness and flavor intensity of the tea soup. The decrease in leachate content at the end of pile fermentation may be attributed to the degradation of macromolecules under microbial and moist heat conditions in the early stages of fermentation. In the later stages, the decline in small soluble substances is due to their consumption by microorganisms [29,30]. However, tea polyphenols may undergo oxidative polymerization under the action of microorganisms and enzymes (polyphenol oxidase), forming theaflavins (TFs), thearubigins (TRs), and theabrownins (TBs). Alternatively, some polyphenolic compounds may be directly metabolized and utilized by microorganisms, leading to a decrease in the total content of tea polyphenols [31,32]. The decrease in free amino acids is closely related to the action of microorganisms. Amino acids serve as a nitrogen source for microbial growth and can be consumed by *Aspergillus niger*, yeast, and others during the pile fermentation process. Furthermore, some amino acids (such as theanine) are deaminated and degraded under high-temperature and high-humidity conditions [33]. The reduction in soluble sugar content may be attributed to its consumption by microorganisms as a carbon source [12]. However, some studies have shown that the soluble sugar content in RpPT increases after pile fermentation, as microorganisms produce enzymes that can degrade cellulose and pectin in the tea cell walls, resulting in the formation of soluble sugars [17,34]. Sugars serve as the primary energy source for microorganisms (such as yeast) and are heavily consumed during fermentation. The content of flavonoids varies differently among samples, showing an increase in the DF, TF, and SG samples but a decrease in the HK samples. This discrepancy may be attributed to factors such as raw materials, fermentation microbial communities, and process parameters (temperature, humidity, time) specific to each sample [35,36,37]. Among TFs, TRs, and TBs, the increase in TBs was the most significant, with relative content ranging between 4.71 and 6.21%, followed by thearubigins, with content levels of 0.62–1.37%; the increase in TFs was minimal, with the highest level barely reaching 0.1%. The increase in TFs is likely attributed to the progressive oxidation of catechins during fermentation [38]. The slight increase in TFs is likely due to their conversion into TBs during fermentation [7]. Furthermore, this substantial increase in TBs can be attributed to the continuous oxidation and polymerization of catechins and their derivatives [7].

To compare the physicochemical characteristics of the four fermentation systems, principal component analysis (PCA) was performed using all measured quality indicators. PC1 and PC2 explained 80.91% and 7.10% of the total variance, respectively, with a cumulative contribution of 88.01%, indicating that the selected parameters effectively represented the major variation among samples (Figure S6). The four fermentation systems exhibited distinct distribution patterns in the PCA score plot, indicating differences in their physicochemical profiles. The separation was mainly driven by PC1, suggesting that the fermentation system was the primary source of variation. Overall, PCA provided multivariate evidence supporting differences among the four fermentation systems beyond comparisons based on individual physicochemical indicators.

Catechins are the primary contributors to astringency in tea infusions [39,40]. Total catechins (TC; TC = GCG (Gallocatechin Gallate) + CG (Catechin Gallate) + ECG (Epicatechin Gallate) + EGCG (Epigallocatechin Gallate) + EGC (Epigallocatechin) + GC (Epicatechin) + C (Catechin) + EC (Epicatechin)) showed a significant decrease after fermentation (*p* < 0.05, Table S1, Figure S1b) [41]. This finding is similar to the results reported by Yang et al. (2025) [14]. The variation trend of CG among the eight catechins was inconsistent across the samples, while the remaining catechins showed a decreasing trend in all samples (Table S1; Figure S1b). GA is an indicator of fermentation degree in RpPT [40]. In the samples of SG and TF, the content of GA showed an increasing trend compared to raw tea, especially in SG, where it reached 2.78 mg/g (*p* < 0.001); however, the GA content decreased in the DF and HK samples (Table S1; Figure S1b). This change could be attributed to the decomposition of catechins, particularly EGCG, into EGC and GA by tannase, which elevates GA [42]. The decrease in GA may also be due to microbial degradation or its involvement in synthesizing TBs [43].

The contents of water extracts, tea polyphenols, free amino acids, soluble sugars, C, EC, ECG, EGC, EGCG, GCG, quercetin, rutin, and luteolin were significantly decreased (*p* < 0.05) in RpPT, whereas flavonoids, theaflavins, thearubigins, and theabrownins were significantly higher (*p* < 0.05). Compared to raw Pu-erh tea, the levels of taxifolin, caffeine, kaempferol, myricetin, GA, and catechin components (CG, GC) showed different changing trends across all fermented samples. These changes in chemical components are likely crucial for the flavor formation of RpPT and contribute to differences in flavor and quality among various types of RpPT.

### 3.2. Changes in Sensory Characteristics

Raw Pu-erh tea exhibits a greenish-yellow or yellow liquor color, with a bitter and thick taste accompanied by a fresh aroma. The samples of DF and HK demonstrated pronounced sourness and astringency. After fermentation, the tea infusion color turns bright orange-red or bright deep red (Figure S2). Compared to raw Pu-erh tea, RpPT exhibits reduced astringency and presents a mellow, sweet, and smooth taste profile with a lingering sweet aftertaste. This is consistent with the commonly observed sensory characteristic changes during the fermentation process of RpPT [44]. The comparative analysis of four samples revealed that TF, SG, and HK showed no significant differences in appearance, but all demonstrated significant differences when compared with DF. In terms of aroma and taste, the HK samples exhibited significant differences compared to the other three samples. However, no significant differences were observed in infused leaves among all samples. Additionally, flavordb was employed to analyze and compare the sensory flavors of the overall substances. The content of the top ten flavor compounds was generally similar across all samples, but some differences were observed. For instance, the levels of floral, woody, and fatty flavors were significantly higher in TF fermentation compared to the other three samples. Overall, in unfermented tea leaves, as tea polyphenols and chlorophyll have not yet undergone oxidative degradation, the aroma of a sample is predominantly characterized by grassy and fresh green notes. The tea liquor appears yellow-green or greenish-yellow and exhibits a certain degree of astringency and bitterness. After fermentation, under the combined effects of warm, humid conditions and microbial activity—along with the catalytic action of metabolic enzymes—compounds such as polyphenols, amino acids, and polysaccharides gradually undergo transformation. Concurrently, the color of the tea liquor shifts to orange-red, bright red, or deep red, while the aroma becomes increasingly rich and complex, developing distinctive flavor characteristics [45].

Electronic nose (E-nose) technology is widely used to discriminate among tea aromatic differences rapidly [46]. Our research found that there are differences among various tea samples in terms of dry tea, tea soup and leaf base. In terms of dry tea, the response values of W5S, DF and HK, as well as TF and SG, were the same, but there were differences between the two groups (Table S2). The response values of W2S in TF are different from those in the other three samples. In terms of soup color and leaf base, the response values of the four samples at W2S, W1W and W1S are different, while others are basically the same. These differences may be the main reasons for the variations in taste and quality among the four fermented samples.

### 3.3. The Dynamic Changes in the Composition of Fungal and Bacterial Communities During the Fermentation Process

We conducted metagenomic sequencing on 75 samples obtained from four distinct fermentation lines across three geographic locations, and we recovered a total of 9,921,386 sequence data points encompassing bacterial and fungal populations, with an average sequence count of 68,443 and 63,843, respectively. In order to better understand the changes in microbial community composition during fermentation, all sequences were clustered according to 97% similarity and annotated; a total of 3421 operational taxonomic units (OTUs) were identified from all samples, of which 1439 OTUs belonged to the fungal domain and 1982 OTUs to the bacterial domain.

**Figure 1:**
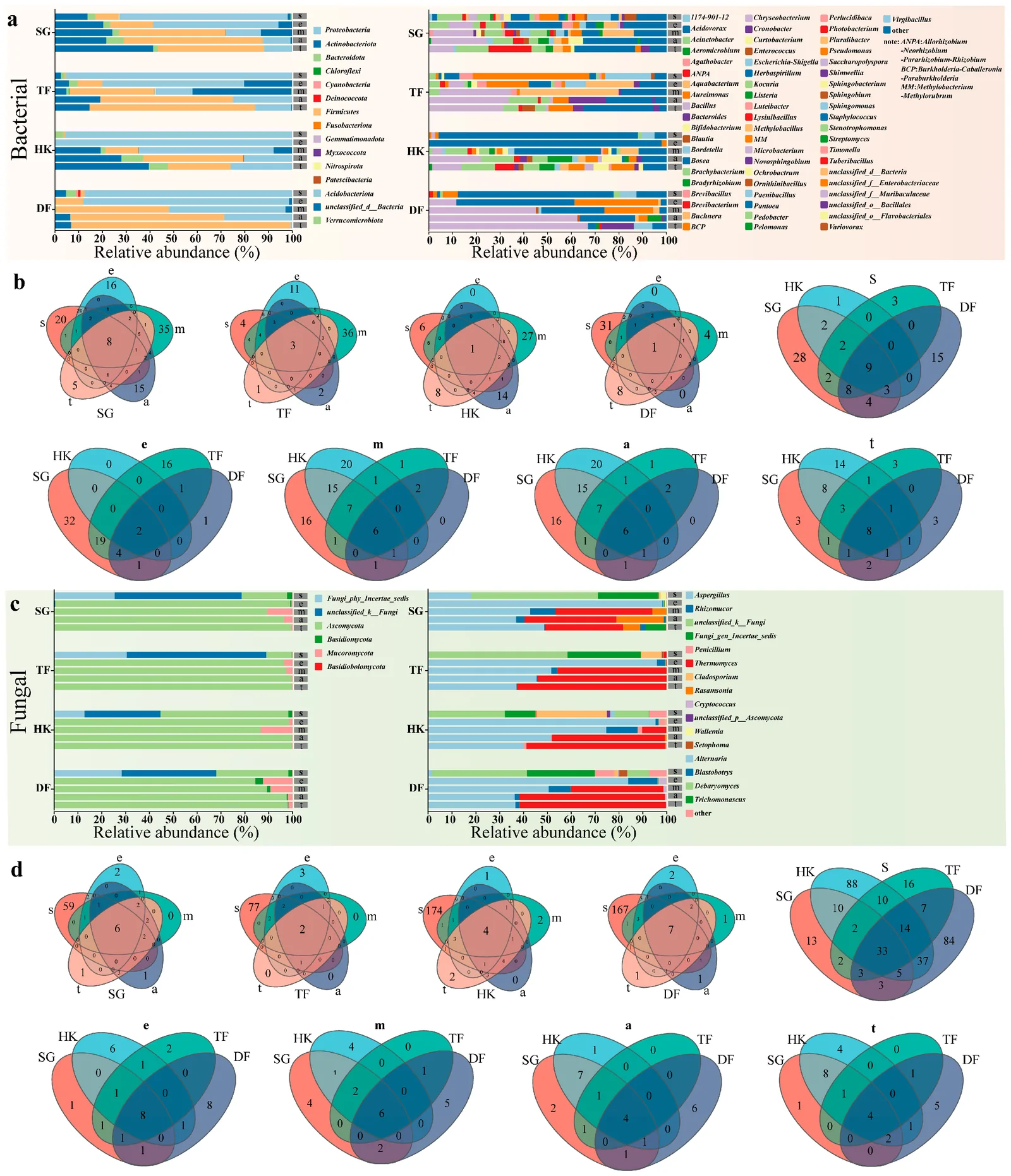
Community composition of dominant microbial phyla and genera at different stages of the fermentation of RpPT. (**a**) The dominant phylum and genera. (**b**) The common and unique bacteria in different fermentation production lines and in different fermentation production lines at the same fermentation stage. (**c**) The dominant fungal phylum and genera. (**d**) The common and unique fungi in different fermentation production lines and in different fermentation production lines at the same fermentation stage.

The bacterial communities in the whole fermentation process were analyzed, and a total of 12 phyla, 120 families, and 221 genera were identified across the 25 samples (Figure 1a,b). The major phyla were Proteobacteria, Firmicutes, and Actinobacteriota; in the early fermentation stage, the proportion of Proteobacteria was up to 15–100%. However, the relative abundance decreased significantly in the late fermentation period to 0.6–28.52%. The trend of Firmicutes was opposite to that of Proteobacteria. In the early fermentation stage, the relative abundance of Firmicutes was relatively low, ranging from 0 to 18.02%, while in the late fermentation stage, the relative abundance of Firmicutes significantly increased, ranging from 13.81 to 92.49%. Actinobacteriota was the most abundant in the Menghai fermented samples, while its relative abundance was low in the TF and DF samples. The difference in Actinobacteriota may be caused by the difference in fermentation environments between the two places. At the genus level, Pantoea had the highest relative abundance in the fermentation samples of HK and DF, especially in the early fermentation period when the relative abundance reached 97.89%. Similar results have also been discovered in previous studies [14]. With an increase in fermentation time, Bacillus gradually became the dominant strain in late fermentation, with a maximum relative abundance of 63.66%. The relative abundance of Staphylococcus was also significantly higher in the SG and TF fermentation samples at the end and in the final samples than in the HK and DF fermentation samples, and its abundance ranged from 19.52 to 29.13%. In addition, analysis of changes in the number of species over time at the genus level showed that, except for DF, the number of species under the other fermentation conditions first increased and then decreased as fermentation progressed, reaching the highest level in the middle stage of fermentation (Figure 1b). Analysis of the four different fermentation samples at the same fermentation stage showed that only approximately 2.63–16% of the genera were shared among the four samples (Figure 1b). These results reveal that different raw materials and fermentation environments can lead to variations in the composition of bacterial communities during fermentation.

The fungal community composition of the different samples was further analyzed (Figure 1c,d). In all the fermentation samples, Ascomycota (84.10–99.95%) dominated with high relative abundance, followed by Mucoromycota (0.2–13.11%). Notably, Ascomycota belonged to the most dominant species throughout the fermentation process, while the abundance of Mucoromycota fluctuated during fermentation, showing decreasing relative abundance with increasing fermentation time. At the genus level, Aspergillus remained the dominant genus throughout the entire fermentation period, particularly in the early stages, with a relative abundance of 83.94–98.34%; subsequently, as the fermentation progressed, its relative abundance gradually decreased. Saccharomyces and Aspergillus are crucial microorganisms in the fermentation process of RpPT, promoting the production of alcohols that contribute to its unique floral and fruity aromas [47].

During the mid-to-late stages of fermentation, the relative abundance of Thermomyces gradually increased, reaching its highest level (32.79–62.59%) by the end of fermentation (Figure 1c). Thermomyces is also a key species in the formation of the flavor of RpPT. Previous research has found that it has a positive correlation with aldehydes [48]. Analysis of fermentation stages showed that fungal diversity peaked initially and then declined significantly across all batches. Across different batches at the same fermentation phase, the proportion of shared microbial species ranged from 10.09% to 24%, indicating that differences in microbial composition were due to variations in raw materials and fermentation conditions (Figure 1d). Nevertheless, each phase maintained its own core microbial taxa. Further analysis demonstrated that Aspergillus, Thermomyces, and Rhizomucor were common across all fermentation stages, while Debaryomyces was shared between the early and mid-to-late stages, and Cladosporium was present in both the early and middle stages. These shared microorganisms likely constitute the core functional microbiota during RpPT fermentation, and their stable presence across samples implies persistent roles in driving critical biochemical reactions (e.g., polyphenol oxidation, glycoside hydrolysis) [14,44,48].

### 3.4. Microbial Community Diversity and Structure Changes During the Fermentation Process

**Figure 2:**
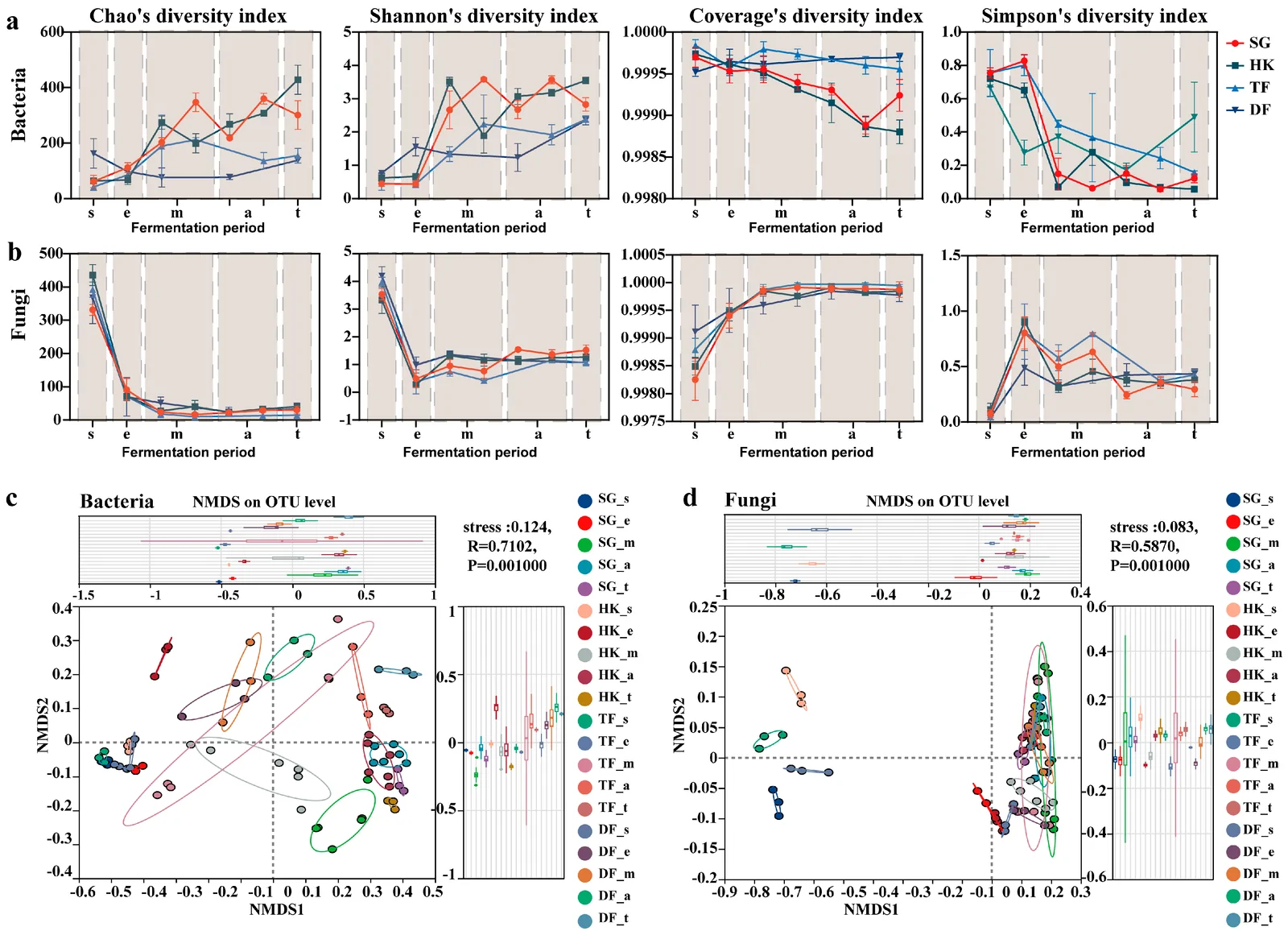
Diversity of microbial communities and analysis of community differences based on NMDS during the fermentation process of RpPT. (**a**) The dynamic changes in bacterial and fungal diversity during the fermentation process of RpPT. (**b**) The dynamic changes in bacterial and fungal community structure during the fermentation process of RpPT. (**c**,**d**) NMDS analysis of bacterial (**c**) and fungal (**d**) communities at the OTU level in different fermentation samples.

To better explore the diversity and dynamics of the microbial communities at different stages of the fermentation period, the α-diversity metrics and β-diversity of the various samples were studied (Figure 2). The results indicated that the α-diversity indices show a general pattern in all fermentation samples. Studies on species richness of bacterial communities found that overall species diversity increased with fermentation time, and the highest species richness was found at the end of fermentation (Figure 2a). However, the opposite trend was found in fungal communities. Namely, the maximum community richness was observed at 0 day, which decreased significantly in the early fermentation stage and then remained relatively stable (Figure 2b). In addition, the bacterial community diversity of DF fermentation was significantly higher than that of SG, HK and TF fermentation in the early stage but was only significantly different from SG and HK in the late stage. At the same time, we found that the geographical locations of SG, HK, TF and DF fermentation were the closest, and the diversity of bacterial communities between them was also the smallest, indicating that microorganisms in the environment may be the key factor leading to the diversity of fermentation bacterial communities. To further assess microbial community diversity changes across the fermentation samples, the Shannon index was compared using the Kruskal–Wallis H test. At the early stage of fermentation, there were no significant differences in fungal communities except for the samples of DF and HK. However, at the middle and late stages of fermentation, there were significant differences in the fungal communities of all samples. After fermentation, the diversity of fungal communities in the SG samples was significantly higher than that in the other samples. The results showed that under different fermentation conditions, the fungal communities showed significant differences in the late fermentation period.

To enhance our understanding of the variations in microbial community structure across samples, the microbial community structures were analyzed using NMDS based on Bray–Curtis distances, and ANOSIM was applied to assess the significance of group separation (*p* < 0.05) (Figure 2c,d). The results showed a significant difference (*p* = 0.001) between the bacterial community compositions of all fermentation samples. Interestingly, the bacterial communities showed a discrete distribution along the NMDS1 axis in correlation with fermentation time, reflecting increasing divergence among communities as fermentation progressed. Figure 2d shows the significant differences in fungal community variation, especially in the samples at the beginning of fermentation. The bacterial and fungal communities exhibited completely distinct succession patterns (Figure 2c,d). The bacterial community showed ongoing succession and increasing structural divergence over time. In contrast, the fungal community shifted abruptly at the onset of fermentation but exhibited slow succession thereafter, with its position on the NMDS1 axis consistently remaining between 0 and 0.2.

### 3.5. Analysis of Species Differences and Interaction Patterns in Microbial Communities

**Figure 3:**
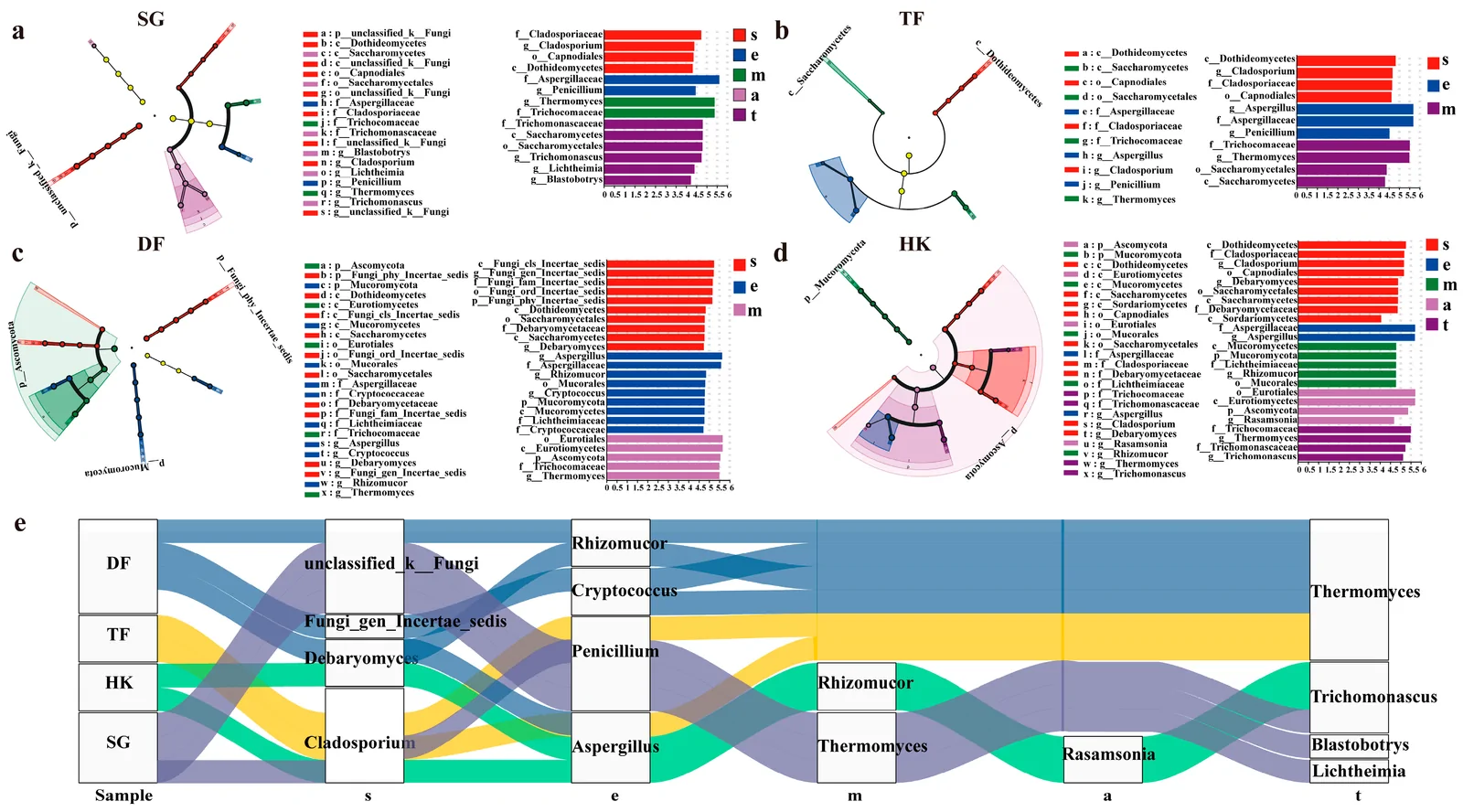
Differences in (**a**–**d**) and succession of (**e**) fungal taxa at different fermentation stages.

The fermentation of RpPT is a complex process involving changes in microbial communities and interactions, and these changes may affect the quality of the tea [49]. The differential microbial taxa across different fermentation stages were analyzed using LDA and LEfSe, considering the LDA score (log10) with a cut-off of 2.0 and a *p*-value < 0.05. The analysis revealed that four fungal biomarkers (Cladosporium, Debaryomyces, unclassified_k__Fungi, and Fungi_gen_Incertae_sedis) were significantly enriched in the s stage (Figure 3). Cladosporium was present in TF, HK and SG, indicating the presence of some fungi from fresh leaves and the environment in the raw materials of sun-dried green tea (Figure 3a–c). There was a significant enrichment of Debaryomyces in the fermentation raw materials of HK (Figure 3d). During the early fermentation stage, Aspergillus exhibited significant enrichment in all samples except SG. In contrast, Penicillium was predominantly enriched in the SG and TF samples. In the DF sample, Cryptococcus and Rhizomucor also showed significant enrichment. The significant enrichment of multiple microorganisms in the early stage might be an important reason for the shortening of the DF fermentation time (Figure 3e). During the m phase, significant enrichments of Thermomyces and Rhizomucor were observed in SG and HK, respectively. However, in the later stages of fermentation, a notable enrichment of Rasamsonia was detected exclusively in the HK fermentation samples. By the final fermentation stage (t phase), Trichomonascus was significantly enriched in both DF and TF, while Thermomyces was prominently enriched in SG and HK. Additionally, Lichtheimia and Blastobotrys were found to be significantly enriched only in the SG samples. These results indicate that the significantly enriched fungi varied across different fermentation processes due to differences in fermentation samples and environmental conditions. However, a general pattern was identified: Aspergillus was consistently the dominant and significantly enriched microbial community in the early stages of fermentation.

At the bacterial phylum level, significant differences in Cyanobacteria, Bacteroidota, Firmicutes, and Actinobacteriota were observed among all samples (Figure S3). Cyanobacteria were significantly enriched in the s and e stages, while Bacteroidota was significantly enriched in the m and a stages. Actinobacteriota and Firmicutes were enriched in the a stage. Additionally, Proteobacteria were significantly enriched in the e stage of the DF and HK samples. At the genus level, the number of significantly different genera gradually increased with fermentation time, reaching a peak in the m stage. Meanwhile, the significantly different genera were largely distinct among different fermented samples at the same stage. Given that the fungal types were generally consistent among different fermented samples at each stage, it was hypothesized that differences in bacterial communities may be a key factor contributing to variations in the quality of final fermented products among different regions. In the e stage, the genera showing significant differences were identified as Pantoea, Pseudomonas, Acinetobacter, Sphingomonas, and Methylobacterium–Methylorubrum. In the m stage, bacterial markers shifted to Shimwellia, Mycobacterium, Kocuria, Bacillus, Thermobacillus, and 33 other genera. A total of 26, 12, and 19 marker genera were identified in stages a, m, and t, respectively. Among them, Brachybacterium, Staphylococcus, Tuberibacillus, and Pluralibacter were markers in stage a, while Enterococcus, Pseudomonas, and Pseudactinotalea were markers in stage m. Notably, Kocuria was significantly enriched across stages m, a, and t, although the specific stages of enrichment varied among samples. A similar pattern was observed for other markers, such as Curtobacterium. These variations may be attributed to differences in fermentation temperature and duration among regions.

The dominant microbial taxa identified in this study may play important ecological roles during ripened Pu-erh tea fermentation. Pantoea, which dominated the early fermentation stage, has been reported to be associated with fresh tea leaves and to preferentially grow at relatively low temperatures, explaining its gradual decline as pile temperature increased [15]. In contrast, Bacillus became dominant during the later stages and is considered one of the core functional bacteria involved in polysaccharide degradation and flavor formation through the secretion of extracellular enzymes [50,51]. Among fungi, Aspergillus remained the predominant genus throughout fermentation and has been reported to participate in carbohydrate degradation, amino acid metabolism, and polyphenol transformation, thereby contributing to the characteristic color, aroma, and taste of ripened Pu-erh tea [50,52]. The continuous increase in Thermomyces during the late fermentation stage is consistent with its thermophilic characteristics and suggests an important role in maintaining metabolic activity under high-temperature fermentation conditions [15,44]. Collectively, these dominant microorganisms are likely to function cooperatively rather than independently, with microbial interactions promoting the sequential transformation of tea constituents and ultimately contributing to the formation of ripened Pu-erh tea quality.

**Figure 4:**
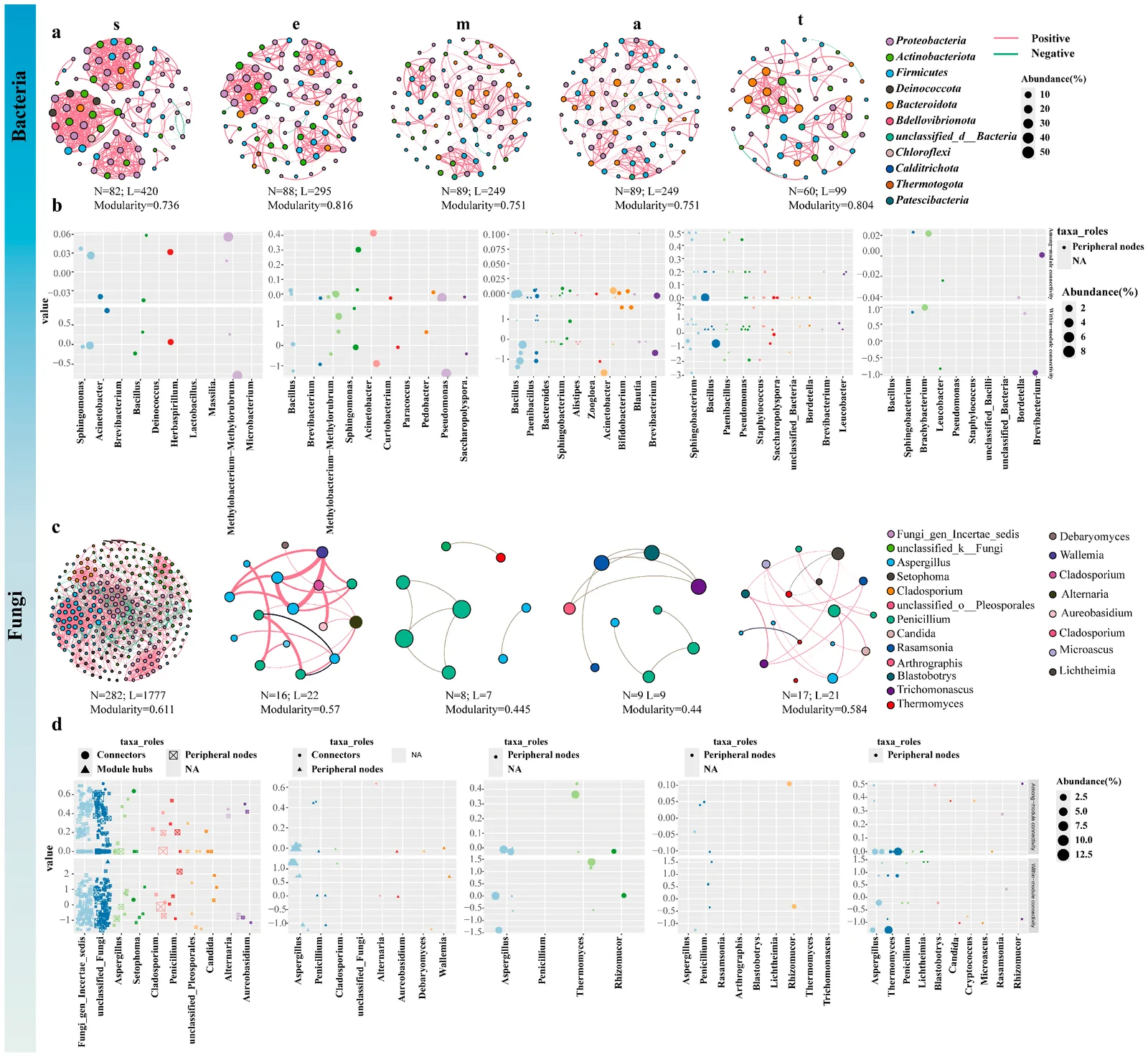
Succession of microbial communities at different fermentation stages. Visualization of networks in bacterial (**a**) and fungal (**c**) groups. A connection represents a significant abs(r) > 0.6, (*p* < 0.05), and significant positive and negative correlations are represented by red and green links, respectively. Different bacterial taxa are shown in different colors. The numbers of nodes and edges are displayed at the bottom. Zi-Pi plots highlighting the keystone genera within the microbial networks of bacterial (**b**) and fungal (**d**) groups.

Microbial interactions, as a biotic factor, have been shown to drive RpPT fermentation community succession [44,53], and they are a major cause of community fluctuations in multi-microbial co-fermentation systems [14]. To elucidate potential microbial interactions and identify keystone species in each stage of RpPT fermentation, all samples at the same fermentation stage were pooled to construct networks at the genus level based on Spearman’s rank correlation (|r| > 0.6; *p* < 0.05) [54]. Multiple network topological indicators consistently indicated that the bacterial community co-networks of s, e, m, a, and t were different, but those of the m and s stages were quite similar (Figure 4a). The interactions among bacterial communities evolved across different fermentation stages of RpPT, with the networks stabilizing in the mid-to-late phases. Network topology analysis revealed a significant reduction in both nodes and edges after fermentation (Figure 4a,c), and the same phenomenon was observed in the fungal networks (Figure 4c). The results indicated that the complexity of the microbial community decreased gradually at the end of fermentation, which may be related to the microbial available substances in tea at the later stage of fermentation [3]. Furthermore, we also found that the ratio of positive correlations among microbial communities was significantly greater than negative correlations throughout the fermentation process (Figure 4a,c). Positive correlations predominated in the bacterial and fungal co-occurrence networks at each stage of RpPT fermentation (Figure 4a,c), consistent with previous reports on the fermentation of Baijiu [55]. Positive correlations dominated all co-occurrence networks, suggesting potential coexistence of microbes through commensalism, cooperation, mutualism, or syntrophy [56], which likely contributes to the stability of the microbial community structure [57]. Positive correlations likely represent mutual symbiosis and predation, whereas negative correlations reflect resource competition [58]. It could be inferred that the members of the microbial community showed more cooperation in the fermentation process.

Keystone taxa, which play an important role in microbial co-occurrence networks, and their changes can lead to changes in microbial community structure and function [59]. According to the node properties of the network, Proteobacteria and Actinobacteria were dominant keystone nodes in each fermentation stage (Figure 4a,b), consistent with the dominant phyla during the pile fermentation of RpPT [21]. In stage s, the number of keystone species was the highest. Besides the above two, there were also Deinococcota, Chloroflexi, Bdellovibrionota, Bacteroidota and Firmicutes. Among them, Bacteroidota was also a keystone species at the e, a and t nodes; Firmicutes was a keystone species in the m and t stages (Figure 4a). In addition, based on the within-module connectivity (Zi) and between-module connectivity (Pi) of nodes in the network, we identified Ornithinibacillus (Zi = 2.97) as a keystone species in the m stage, while unclassified_f__Rhizobiaceae (|Zi| = 2.82) and Sphingobacterium (|Zi| = 2.62) were recognized as keystone species in the a stage (Figure 4b). For the fungal community, Ascomycota was identified as the core fungal taxon throughout the entire fermentation process. Additionally, Basidiomycota was determined to be a key taxon during the s and e stages, while unclassified_k__Fungi and Mucoromycota were recognized as crucial taxa in the s stage and t stage, respectively (Figure 4b). In s stage, a few fungi were found to act as connectors (i.e., nodes that highly connect modules), and they mainly belonged to unclassified_k__Fungi, Fungi_gen_Incertae_sedis and Setophoma. On the other hand, one module hub (highly connected node within modules) was only detected in the s stage network, and it belonged to unclassified_k__Fungi. The keystone species of Alternaria were found to act as connectors in the e-stage networks (Figure 4d).

The topological indices of co-occurrence networks are key indicators of their structure [60]. The modularity indices of bacterial and fungal co-occurrence networks exceeded 0.44 (Figure 4a,c), suggesting a well-organized and functionally interrelated modular structure in FD microbial communities. High modularity enhances network stability by localizing disturbances within modules, preventing them from spreading and allowing the network to maintain its functions despite environmental changes [61]. Furthermore, the number of nodes and edges decreased significantly as fermentation progressed, a trend observed in both bacterial and fungal networks. This indicates a gradual reduction in the complexity of microbial community interactions during RpPT fermentation. Although cooccurrence network analysis provides a system-level overview of potential interdependencies among microorganisms, accurate results still require experimental verification. 

### 3.6. The Functions of Bacterial and FUNGAL Communities

**Figure 5:**
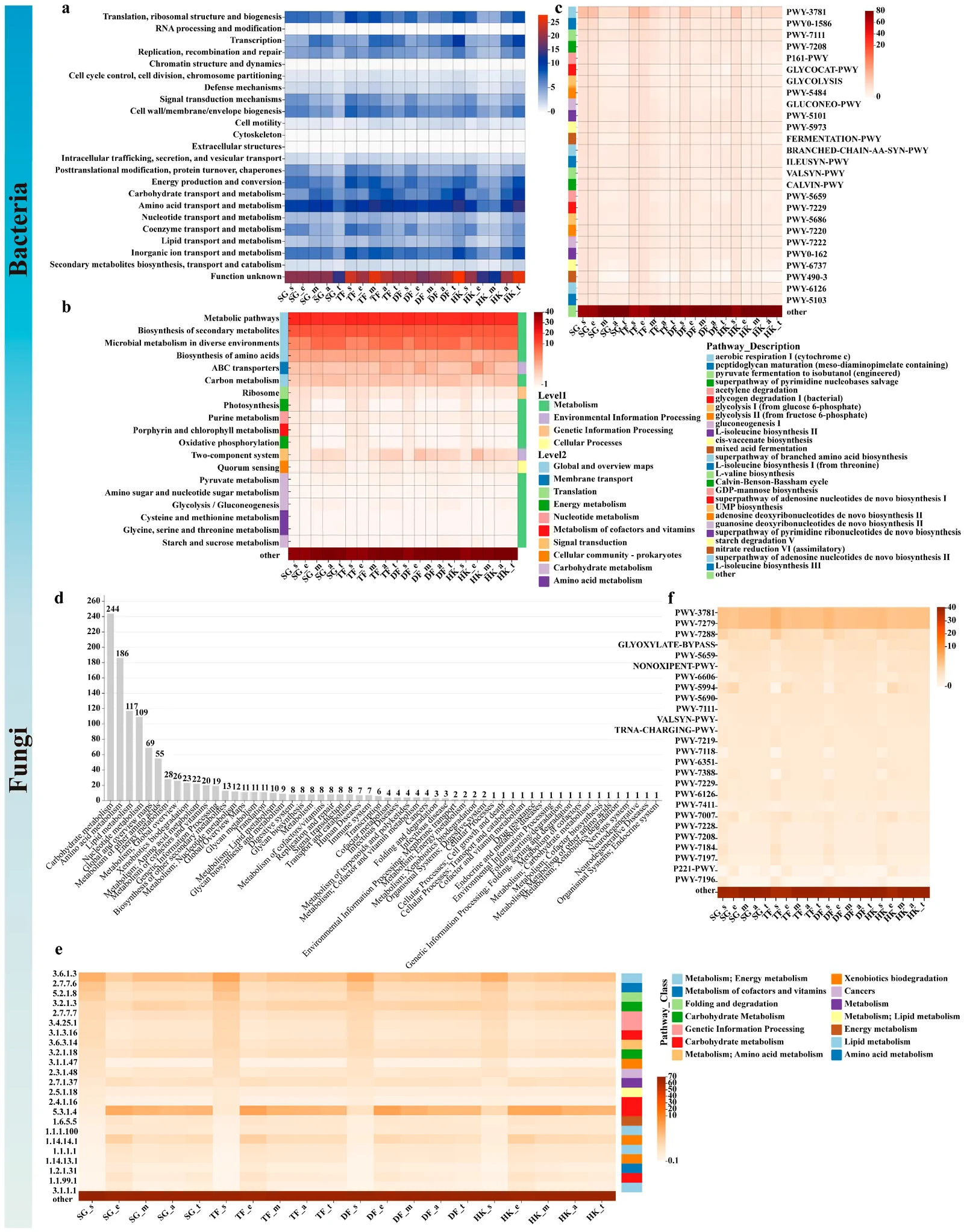
The functions of bacterial and fungal communities in different RpPT fermentation samples. (**a**) Metabolic functions involving bacterial communities. (**b**) Distribution of KEGG functional abundances in different samples. (**c**) Metabolic pathways of bacteria in each sample. (**d**) Metabolic functions of fungal communities in each sample. (**e**) The distribution of enzymes with a relative abundance greater than 1% in each sample. (**f**) The metabolic pathways of the fungal communities in each sample.

Microorganisms produce enzymes that catalyze a series of reactions—including oxidation, condensation, degradation, and polymerization. These reactions drive the breakdown of macromolecules into diverse flavor compounds, critically shaping RpPT fermentation [62]. PICRUSt2 was applied to predict the functional genes encoding key enzymes in the fermentation process of RpPT. Functional gene annotation was performed using the COG, MetaCyc, and KEGG databases (Figure 5). According to the COG annotation results, the bacterial communities were primarily involved in 22 functional categories, including amino acid transport and metabolism, among others, as well as some function unknown categories (Figure 5a). The relative abundance of function unknown was observed to be the highest, ranging from 12.14% to 27.86%, followed by amino acid transport and metabolism and translation, ribosomal structure and biogenesis, with abundances ranging from 6.44% to 14% and 4.00% to 8.72%, respectively (Figure 5a). It is noteworthy that in all fermentation processes, the functions of amino acid and carbohydrate transport and metabolism were significantly enhanced during the mid-to-late stages. This enhancement effect may facilitate protein hydrolysis and the synthesis of microbial metabolites, which is consistent with previous research results [63]. According to the KEGG annotation, a total of 408 metabolic functions were annotated at the tertiary level, while 46 and six metabolic functions were annotated at the secondary and primary levels, respectively (Figure 5b). The highest functional abundance was associated with metabolic pathways, followed by biosynthesis of secondary metabolites, microbial metabolism in diverse environments, and biosynthesis of amino acids. At the primary level, the largest number of genes was found to be involved in metabolic pathways (Figure 5b). The bacterial communities were annotated using the MetaCyc database, revealing their involvement in 376 metabolic pathways (Figure 5c). In the initial stage (s and e stage), the abundance of most metabolic pathways was observed to be relatively high, followed by a subsequent decrease and maintenance at a stable level (Figure 5c). Notably, the abundance associated with aerobic respiration I (cytochrome c) was found to be particularly elevated, especially during the s and e stages.

Functional profiling of the fungal community during RpPT fermentation, annotated via the KEGG database, identified 876 enzymes primarily involved in carbohydrate, amino acid, and lipid metabolism, as well as global and overview pathways (Figure 5d). Enzymes with ≥1% relative abundance in any sample were further analyzed. Their gene abundance showed consistent trends across all four fermentation batches, differing only in magnitude (Figure 5e). A similar trend was also observed in the top 31 metabolic pathways annotated by the MetaCyc database (Figure 5f). According to the MetaCyc database, fungi were involved in 74 metabolic pathways, with the top 31 analyzed. Pathways including galactose degradation I, NAD/NADH phosphorylation and dephosphorylation, D-galactose degradation V, chitin degradation to ethanol, and palmitate biosynthesis I showed an initially low abundance that increased over time, likely corresponding to the degradation of sugars and other compounds in tea leaves [4,12,29]. Additionally, PICRUSt2-based predictions indicated that inferred metabolic pathways and their abundance dynamics were highly consistent across all four fermentation batches, which may partially explain the uniform taste of the final tea products. The formation of RpPT quality was primarily attributed to the synergistic effects of microbial enzymes (e.g., polyphenol oxidase, cellulase, β-glucosidase) and endogenous tea enzymes [21]. Through reactions such as polyphenol oxidation, polysaccharide degradation, and protein hydrolysis, the characteristic reddish-brown color, mellow taste, and aged aroma were achieved. The quality of RpPT was highly dependent on the fungal-dominated solid-state fermentation process, in which fungal-secreted carbohydrate- and amino acid-metabolizing enzymes played a critical role in the degradation of macromolecules such as polysaccharides and proteins, thereby significantly influencing the color, taste, and aroma of RpPT [21,64]. In this study, numerous enzymes associated with the formation of key aroma and flavor compounds in RpPT were also annotated, including laccase, β-glucosidase, cellulase, pectinesterase, protease, lipoxygenase, and aminopeptidase.

### 3.7. Analysis of Aroma Compound Profiles of RpPT

**Figure 6:**
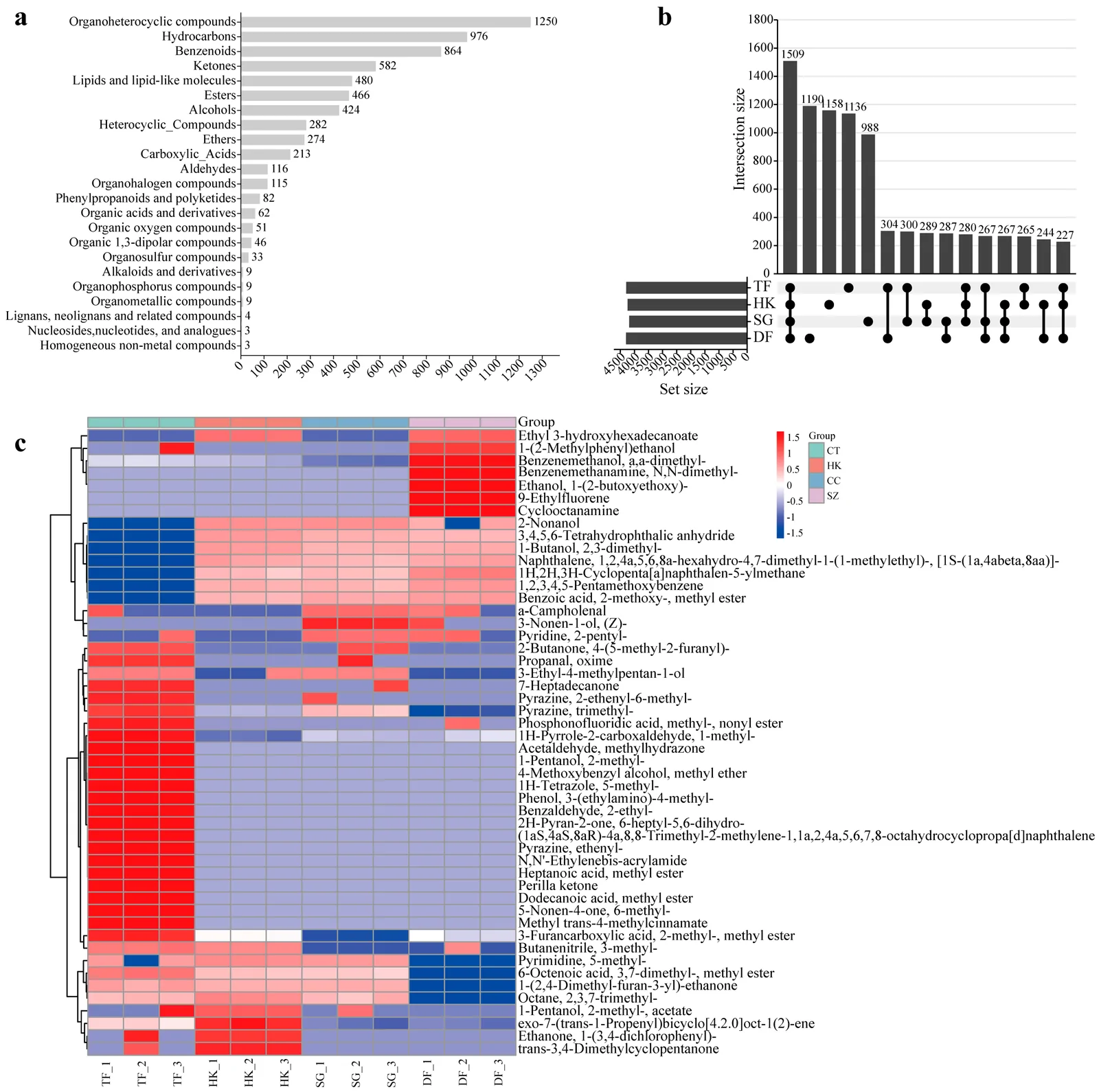
The profile of volatile compounds in different RpPT samples. (**a**) Classification and proportion of volatile substances. (**b**) The number of shared and unique volatile compounds in each sample. (**c**) Comparative analysis of the top 50 volatile substances by abundance in each sample.

Aroma is one of the important factors affecting the quality of RpPT, and it is also a key indicator for evaluating its value [47,65]. A total of 8711 distinct substances were initially identified across the different samples, with 4288 in TF, 4240 in HK, 4186 in SG, and 4295 in DF. The classification of these compounds was achievable for 72.94% (6354) of them, with the highest proportions belonging to organoheterocyclic compounds (19.67%), hydrocarbons (15.36%), and benzenoids (13.60%), followed by ketones (9.16%), lipids and lipid-like molecules (7.55%), esters (7.33%), and alcohols (6.67%) (Figure 6a). Analysis revealed a highly consistent compositional profile in the RpPT samples, dominated by organic heterocyclic compounds, ketones, benzene-ring compounds, lipids, hydrocarbons, esters, aldehydes, alcohols, ethers, carboxylic acids, and other oxygenated organics (Figure 6a). It has been reported that most of these substances contribute significantly to the flavor profile of RpPT [35,44]. Furthermore, although a large number of compounds are shared by all the samples, there are also many compounds that are unique to each sample, and the abundance of many of the common compounds varies among the samples (Figure 6b,c). For instance, homogeneous non-metal compounds, organic polymers, and organic salts were identified. These compounds are suggested to be the key substances responsible for the flavor variations among different RpPT samples [55,64].

To further elucidate the influence of different fermentation systems on flavor formation in ripened Pu-erh tea, Z-score normalization was performed based on key flavor-related metabolites and sensory attributes (Figure S7). The results revealed distinct flavor profiles among the fermented samples. Most key flavor-related metabolites in the TF sample, including pyrazine, benzyl alcohol, nonanal, and methyl eugenol, exhibited higher accumulation levels, which corresponded to its higher aroma, taste, and liquor color scores, suggesting enhanced formation of characteristic aged aroma and mellow flavor compounds during fermentation. The HK sample showed relatively higher levels of aroma-related compounds, such as α-terpineol and benzeneacetaldehyde, which may partially contribute to its specific aroma profile. In contrast, the DF sample exhibited lower levels of most flavor-related metabolites, consistent with its weaker sensory performance. The SG sample displayed a distinct flavor metabolic pattern, characterized by higher accumulation of hexadecanoic acid ethyl ester, which may be associated with its unique taste characteristics. Overall, different fermentation systems resulted in differentiated accumulation patterns of flavor-related metabolites and sensory characteristics, with TF showing a more favorable flavor profile and sensory quality.

To further elucidate the similarities and differences in the different RpPT samples at the molecular level, principal component analysis (PCA), Hierarchical Cluster Analysis (HCA), and Orthogonal Partial Least Squares Discriminant Analysis (OPLS-DA) were employed to analyze the variations in flavor compounds among four fermented samples (Figure S4). The results highlighted significant differences in the chemical composition among the fermented tea samples. OPLS-DA was utilized to construct a model for identifying specific markers with larger contributions. The values of R2X, R2Y, and Q2 in the OPLS-DA model were 0.319, 0.999, and 0.761, respectively, indicating that the model exhibited excellent stability and predictive ability. (The model demonstrated strong discriminative power (R2Y close to 1) and outstanding predictive capability (Q2 > 0.75), although the explanatory rate for the X variables was relatively low, suggesting that only a subset of variables strongly contributed to tea flavor.) To compare the samples and identify key flavor contributors in RpPT, 1509 compounds were found to be common to all four. Based on the abundance and relative odor activity value (ROAV) of these compounds in each sample and the screening criteria of significant differences (VIP ≥ 1 and *p* < 0.05) and ROAV > 1, 2-Nonenal, (E)-, Butanal, 2-methyl-, Heptanal, 2-Undecanone, 2-Octenal, (E)-, 2,3-Butanedione, furan, 2-pentyl-, pyrazine, trimethyl-, 1-Octen-3-one, 2,6-Nonadienal, (E,Z)-, butanoic acid, 3-methyl-, hexadecanoic acid, and ethyl ester were identified as having greater contributions to the flavor profile of RpPT (Figure S4c). To further explore the differences between the samples, pairwise OPLS-DA models were constructed, identifying 618 differential metabolites across the four fermented tea samples (VIP > 1, *p* < 0.05). Overall, based on the above research results, we found that the types and contents of compounds vary among the different samples, which is consistent with previous research results [47]. This might be the reason for the differences in flavor and quality among the different samples.

### 3.8. The Correlation Between Microbes and Characteristic Flavor Metabolites in RpPT

Microorganisms play a central role in the post-fermentation process of RpPT, with their metabolic activities directly determining its quality characteristics. During the pile fermentation stage, microbial communities dominated by *Aspergillus niger*, yeasts, *Penicillium*, and *Rhizopus* secrete extracellular enzymes to decompose cellulose, pectin, and proteins in tea leaves, promoting the oxidative degradation of tea polyphenols, reducing bitterness and astringency, and forming a reddish-brown, translucent liquor [64]. Concurrently, organic acids, esters, and aromatic substances (such as terpenes, aldehydes, and ketones) produced through microbial metabolism contribute to the unique aged aroma, mellow taste, and sweet aftertaste of RpPT [64]. Furthermore, specific microbial communities can generate bioactive compounds (such as theabrownins and gallic acid) through biotransformation, enhancing the tea’s health benefits [44]. To elucidate the relationship between flavor compounds and microorganisms, the relative abundances of microbial genera greater than 1% at each stage or those identified as core microorganisms were selected and correlated with 630 key flavor compounds in this study. The O2PLS model was constructed to evaluate the influence of microbial markers from 38, 26, 47, 35, and 35 genera on 49 key aroma compounds during RpPT flavor formation (Figure S5). During the s stage, the greatest contributions to flavor formation were made by *Cladosporium*, Methylobacterium–Methylorubrum, *Alternaria*, *Pedobacter*, and *Buchnera*. In the e stage, *Streptomyces*, *Kocuria*, *Staphylococcus*, *Rhizomucor*, and *Luteibacter* were identified as the most significant contributors to flavor development. The m stage was characterized by the dominant influence of *Cryptococcus*, *Curtobacterium*, *Perlucidibaca*, and *Acinetobacter* in flavor formation. During the a stage, the primary microbial contributors to flavor were found to be *Shimwellia*, *Brachybacterium*, *Kocuria*, *Bacillus*, and *Enterococcus*. Finally, in the t stage, the most substantial roles in flavor formation were played by *Pseudomonas*, *Shimwellia*, *Virgibacillus*, *Rhizomucor*, and unclassified_o__Bacillales.

**Figure 7:**
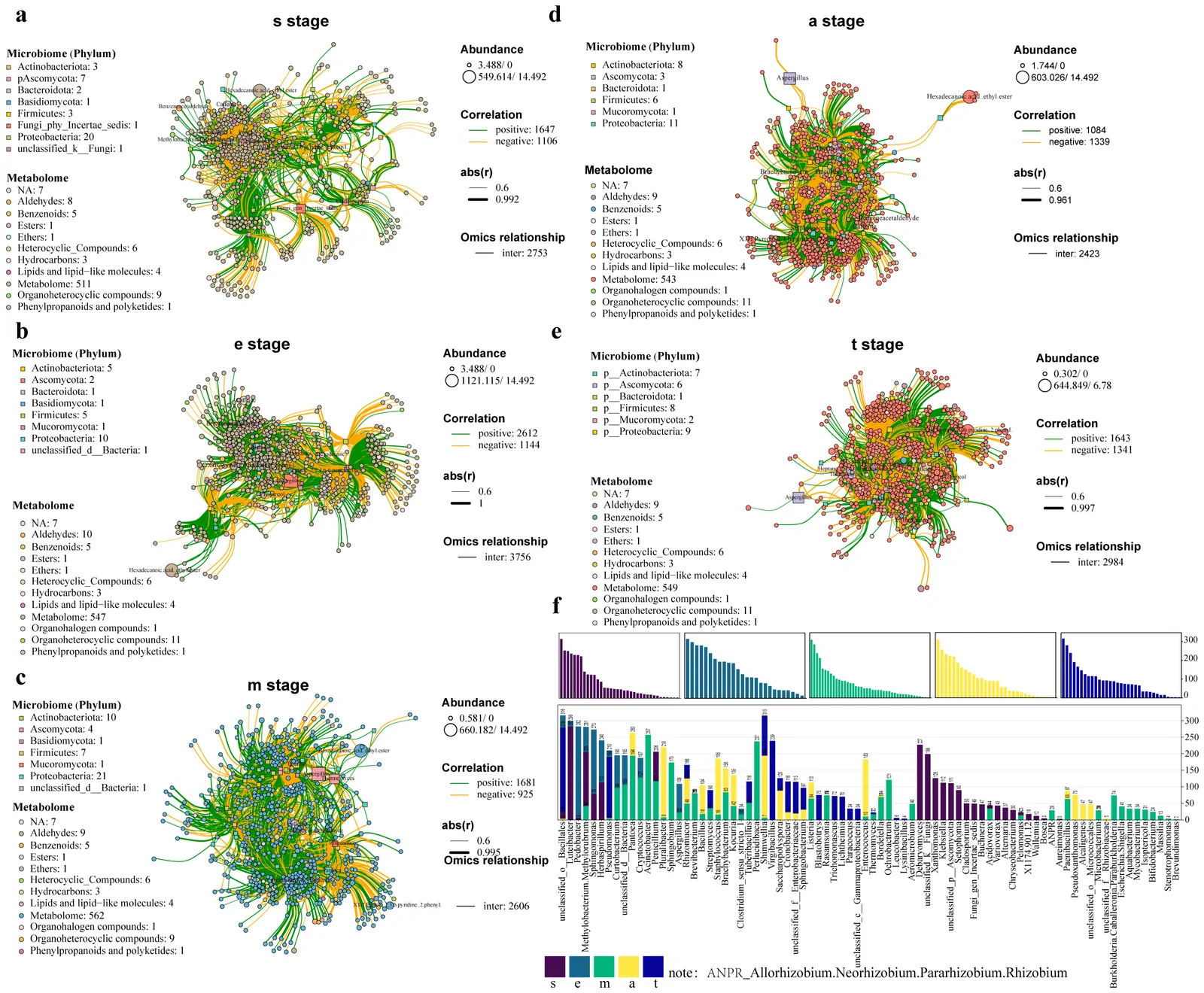
Association analysis between microorganisms and key volatile substances. (**a**–**e**) The association between microorganisms and key volatile substances at different stages of fermentation. (**f**) Variation in the number of microorganisms related to flavor substances at different fermentation stages.

Using Pearson correlation coefficients, a multi-omics network was constructed to analyze microorganism–flavor compound relationships, based on significance (*p*-value) or correlation thresholds (|r| > 0.6) (Figure 7). The results demonstrated that during the s stage, the highest number of flavor compounds was associated with *Luteibacter*, *Debaryomyces*, and *Pantoea*, followed by *Acinetobacter*, *Penicillium*, and *Methylobacterium–Methylorubrum*. Additionally, *Luteibacter* was found to be significantly positively correlated with aldehydes (2,4-Hexadienal, (E,E)-; Furfural), ketones (4-Hepten-3-one, 5-methyl-, (E)-; 1-Penten-3-one), pyrazines (pyrazine, ethyl-), lactones and furans (furan, 2-ethyl-), as well as terpene derivatives and phenols (1,3-Benzenediol, 4-propyl-; Cyclopentanol, 3-methyl-2-(2-pentenyl)-). Notably, *Cladosporium* was observed to exhibit a significant positive correlation with trans-3,4-Dimethylcyclopentanone (r = 0.83; *p* < 0.001), which belongs to cyclic ketones and typically possesses woody, fruity, or caramel-like aromatic characteristics. Furthermore, Methylobacterium–Methylorubrum was identified to be strongly positively correlated with the key tea aroma compound pyrazine, 2-ethyl-6-methyl- (r = 0.89; *p* < 0.001) (Figure 7a). During the e stage, the highest number of compounds was found to be significantly associated with unclassified_o__Bacillales, *Luteibacter*, and *Pedobacter*, followed by Methylobacterium–Methylorubrum, *Sphingomonas*, *Herbaspirillum*, and *Pseudomonas*. Further analysis revealed that unclassified_o__Bacillales, *Luteibacter*, and *Pedobacter* were significantly positively correlated with 252, 259, and 234 types of aldehydes, esters, terpenes, and sesquiterpenes, respectively (Figure 7b). During the m stage, *Acinetobacter*, *Perlucidibaca*, *Pantoea*, *Sphingobium*, *Cryptococcus*, and *Ochrobactrum* were identified as the genera most closely related to flavor compounds. Among them, 192 flavor compounds were significantly positively correlated with *Acinetobacter*, 201 with *Perlucidibaca*, and 40 with *Pantoea*, while 154 were significantly negatively correlated with *Pantoea*. Additionally, *Acinetobacter*, *Perlucidibaca*, and *Cryptococcus* were confirmed as key species in the formation of RpPT flavor through O2PLS analysis (Figure 7c). In the a stage, the highest number of flavor compounds was associated with *Pantoea*, *Pluralibacter*, *Shimwellia*, *Staphylococcus*, and *Enterococcus* (Figure 7d). In the t stage, the most flavor compounds were linked to *Shimwellia*, unclassified_o__Bacillales, *Virgibacillus*, *Pseudomonas*, and *Rhizomucor*, which was largely consistent with the results of the O2PLS analysis, indicating that these microorganisms play a crucial role in the formation of flavor during the later fermentation stages of RpPT (Figure 7e). Yang et al. (2025) also reported that *Pantoea* and *Pseudomonas* were identified as key microbiomarkers that significantly affect key flavor compounds during the MT_RPT flavor degradation process [14]. In addition, we also found that the number of microorganisms related to flavor substances varies at different fermentation stages (Figure 7f). These results suggest that different microorganisms contribute to RpPT flavor formation at different fermentation stages.

It should also be noted that the four fermentation lines differed in several production factors, including raw material origin, pile size, fermentation duration, turning frequency, temperature management, and processing mode (traditional versus digital fermentation). These variables are known to influence oxygen availability, heat accumulation, moisture distribution, and substrate utilization, thereby affecting microbial succession and metabolic activities. Consequently, the observed differences in microbial community composition and tea quality are likely driven by the combined effects of these processing variables rather than by microbial succession alone. Future studies should further control individual processing parameters to clarify their respective contributions to microbial ecology and quality formation.

### 3.9. Correlation Analysis Between Physicochemical Properties and Microbial Communities

**Figure 8:**
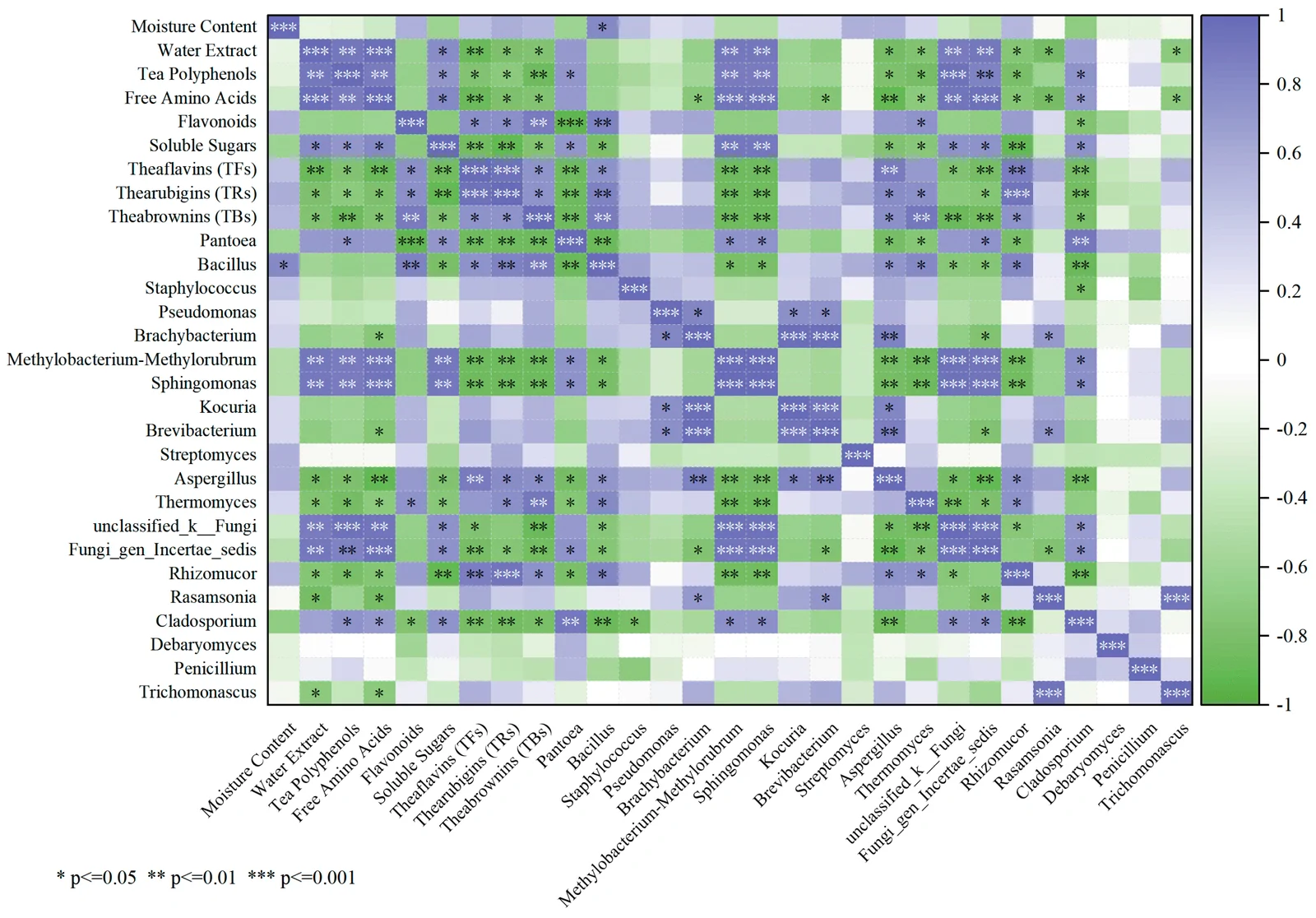
Heatmap of correlations between microorganisms and physicochemical properties during RpPT fermentation.

To further explore the relationships between microbial succession and quality transformation during RpPT fermentation, Spearman correlation analysis was performed between dominant microbial genera and physicochemical properties (Figure 8). Significant correlations were observed between key microorganisms and quality-related components. Among *fungi*, *Aspergillus* was positively correlated with TFs (ρ = 0.894, *p* = 0.003) and negatively correlated with free amino acids (ρ = −0.905, *p* = 0.002). *Thermomyces* was strongly positively correlated with TBs (ρ = 0.913, *p* = 0.002), while *Rhizomucor* was positively correlated with TRs (ρ = 0.936, *p* < 0.001) and negatively correlated with soluble sugars (ρ = −0.903, *p* = 0.002). Among *bacteria*, *Bacillus* was positively correlated with TBs (ρ = 0.884, *p* = 0.004), whereas *Pantoea* was negatively correlated with TFs (ρ = −0.877, *p* = 0.004). These contrasting patterns suggest that microbial succession was closely associated with the depletion of primary quality components and accumulation of tea pigments during fermentation.

Overall, the correlation analysis demonstrated that dominant microorganisms, particularly *Aspergillus*, *Thermomyces*, *Bacillus*, and *Pantoea*, were closely associated with the transformation of key physicochemical components. These results suggested that microbial succession may play an important role in regulating substrate degradation and the formation of characteristic quality attributes during RpPT fermentation.

## 4. Conclusions

In summary, the composition, structure and function of the microbial community, interaction patterns and flavor substances of four RpPT samples were comprehensively investigated, with the key flavor substances being correlated with their corresponding microorganisms. A consistent phenomenon was observed across different fermentation samples: the diversity of the fungal community was significantly reduced, and *Aspergillus* and *Thermomyces* were the most dominant microorganisms in the RpPT fermentation process. The mutual relationships among microorganisms were mainly characterized by positive correlations, indicating that cooperative relationships among microorganisms predominate over competitive ones. The abundance of the flavor compounds varies among the different fermentation samples, but the composition pattern is basically the same. *Pantoea*, *Penicillium* and Methylobacterium–Methylorubrum are among the key microorganisms in the fermentation of RpPT, and there is a significant correlation between them and various flavor compounds. This study reveals the overall patterns of change during RpPT fermentation and provides insights into the potential differences associated with raw materials and fermentation conditions. It provides a basis for optimizing the fermentation process of RpPT and improving its quality. It is worth noting that although amplicon sequencing and predictive functional analyses can effectively predict the potential roles that microorganisms may play in the fermentation of Pu’er tea, further experiments are still needed to conduct in-depth research and verification in the future.

## Data Availability

The original contributions presented in this study are included in this article/the Supplementary Materials. Further inquiries can be directed to the corresponding authors.

## Supplementary Materials

The following supporting information can be downloaded at https://www.mdpi.com/article/10.3390/foods15173162/s1. Supplementary data for this paper can be found in the document named Supplementary: Figure S1. The changes in the characteristic components of tea leaves before and after fermentation of Pu-er tea. (**a**) The basic physical and chemical characteristics of tea; (**b**) Components such as catechins, flavonoids, alkaloids, and phenolic acids. Figure S2. The visual observation of the dry tea leaves, liquid color and tea infusion appearance before and after the fermentation of Pu-er tea. Figure S3. LEfSe analysis and succession of differential bacterial taxa during ripened Pu-erh tea fermentation. (**a**–**d**) LEfSe analysis of bacterial communities in SG (**a**), HK (**b**), DF (**c**), and TF (**d**). (**e**) Sankey diagram showing the succession of differential bacterial genera across fermentation stages. Figure S4. The changes of chemical composition during the fermentation of RpPT. (**a**) Hierarchical cluster and (**b**) principal component (PCA) analysis of samples. (**c**) Orthogonal Projections to latent structures discriminant analysis (OPLS-DA) loading diagram. (**d**) OPLS-DA score plot. Figure S5. O2PLS joint loading plots of flavor compounds (X) and microbial genera (Y) at different fermentation stages. Figure S6. The PCA score plots of the four fermentation production lines, where sample numbered 1 represents Raw Pu-erh and sample numbered 2 represents RpPT. Figure S7. RpPT sensory evaluation score and standardized heatmap of representative metabolites’ Z-scores. Table S1. The changes of physicochemical properties during fermentation of RpPT. Table S2. The E-Nose sensing values of four production lines for RpPT.
