# Supplementary material for: Microbial Succession, Functional Dynamics, and Their Relationship with Quality Formation During Ripened Pu-Erh Tea Fermentation
Source: Foods. 2026 Sep 7;15(17):3162. doi: 10.3390/foods15173162 (PMC13564671; doi:10.3390/foods15173162)
Supplement: Supplementary file 1 [file foods-15-03162-s001.zip › foods-4427203-supplementary.pdf]

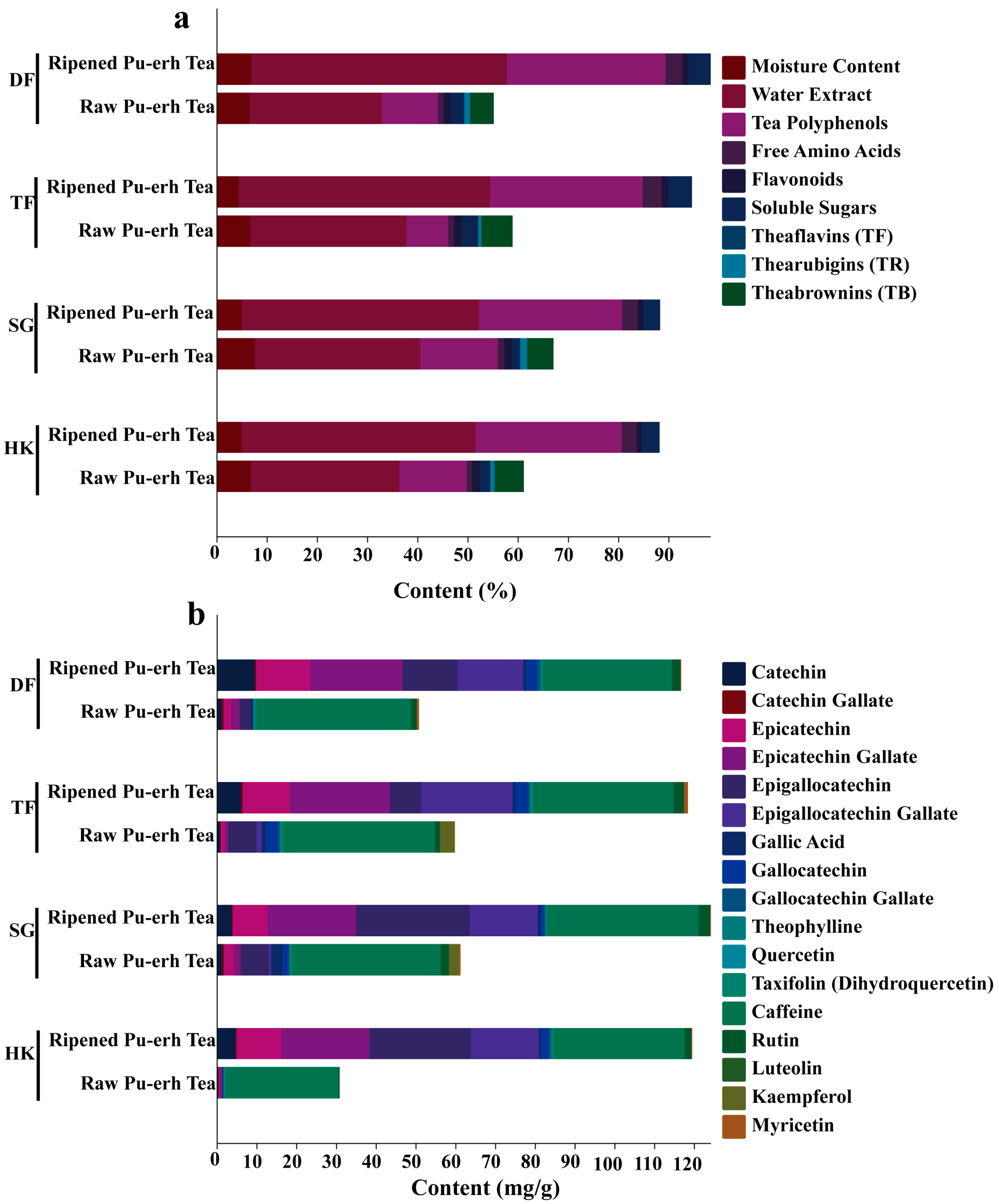

Figure S1 The changes in the characteristic components of tea leaves before and after fermentation of Pu-er tea. **a** The basic physical and chemical characteristics of tea; **b** Components such as catechins, flavonoids, alkaloids, and phenolic acids.

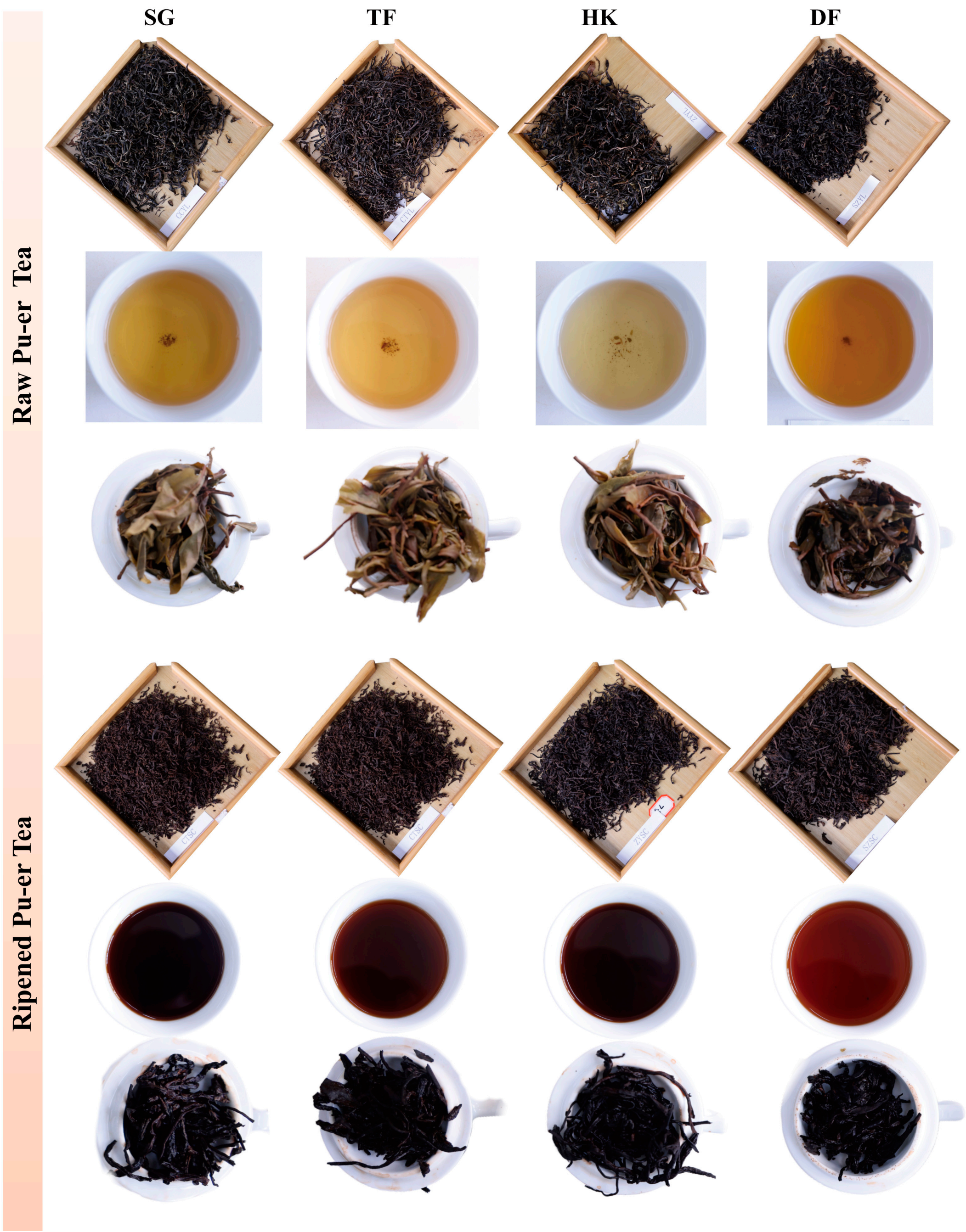

Figure S2 The visual observation of the dry tea leaves, liquid color and tea infusion appearance before and after the fermentation of Pu-er tea.

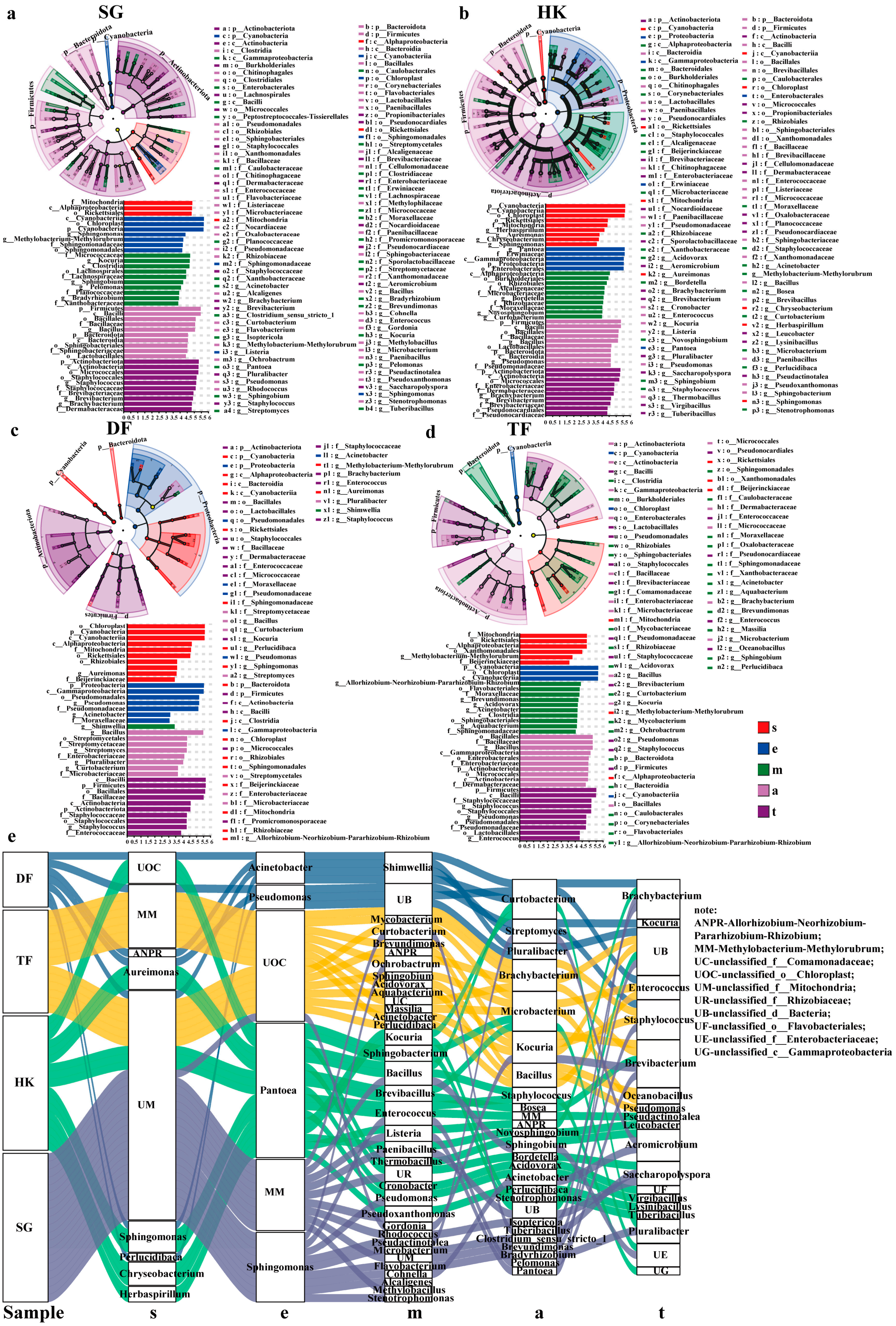

Figure S3 LEfSe analysis and succession of differential bacterial taxa during ripened Pu-erh tea fermentation. (a–d) LEfSe analysis of bacterial communities in SG (a), HK (b), DF (c), and TF (d). (e) Sankey diagram showing the succession of differential bacterial genera across fermentation stages.

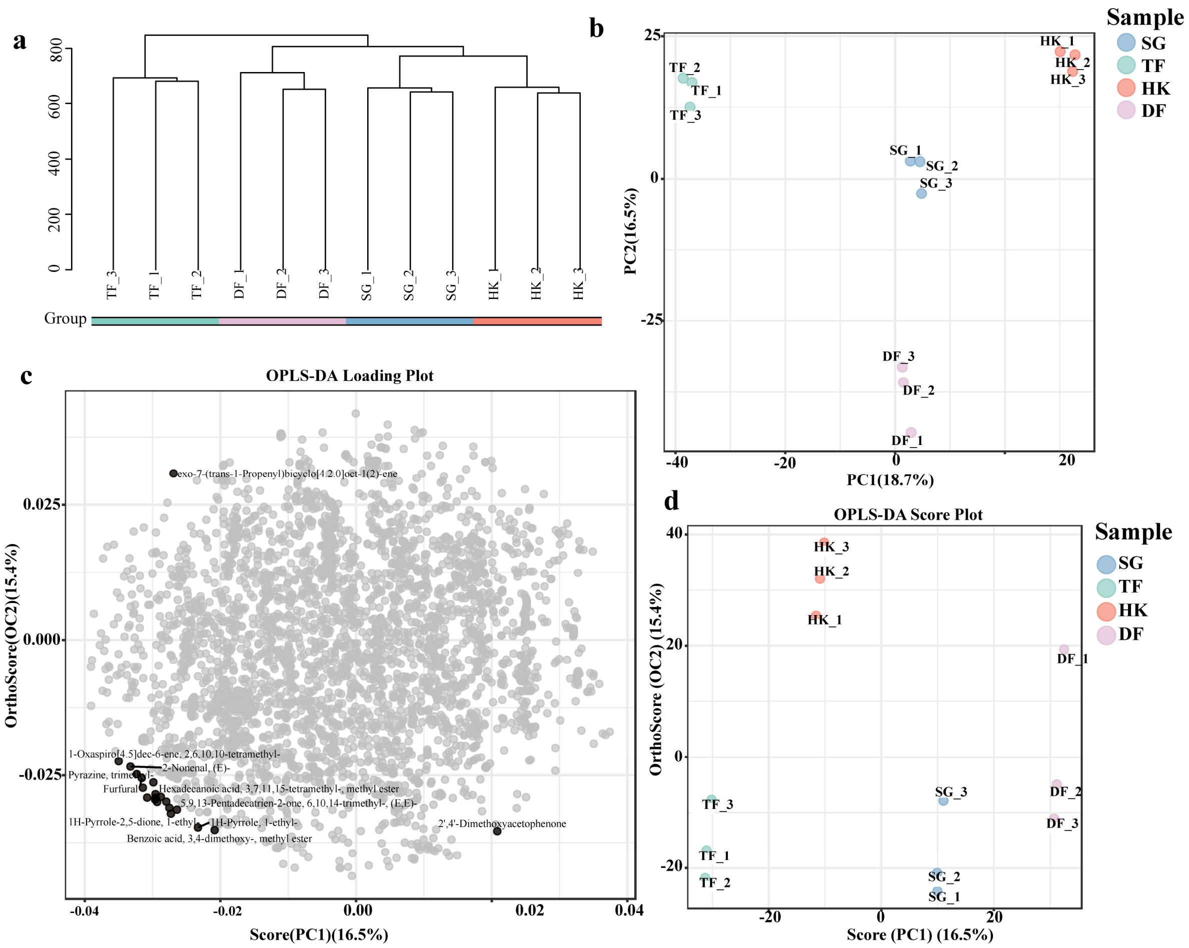

Figure S4 The changes of chemical composition during the fermentation of RpPT. **(a)** Hierarchical cluster and **(b)** principal component (PCA) analysis of samples. **(c)** Orthogonal Projections to latent structures discriminant analysis (OPLS-DA) loading diagram. **(d)** OPLS-DA score plot.

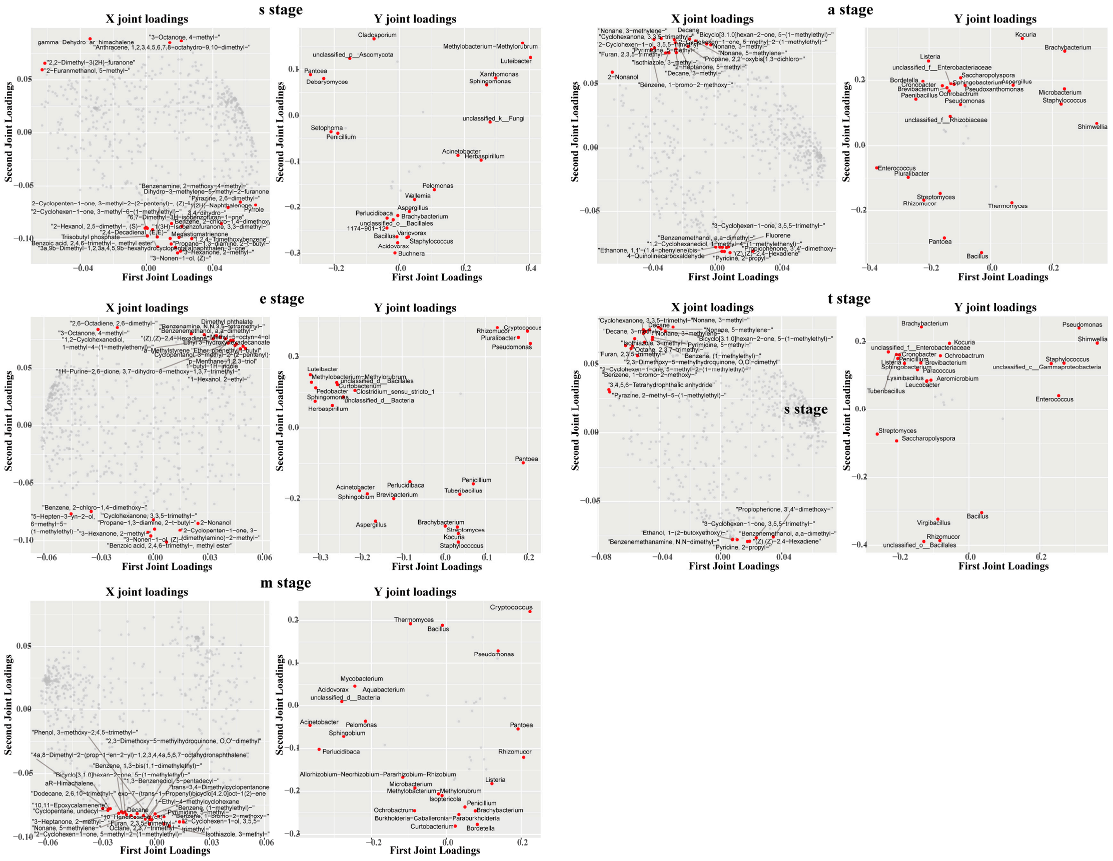

Figure S5 O2PLS joint loading plots of flavor compounds (X) and microbial genera (Y) at different fermentation stages.

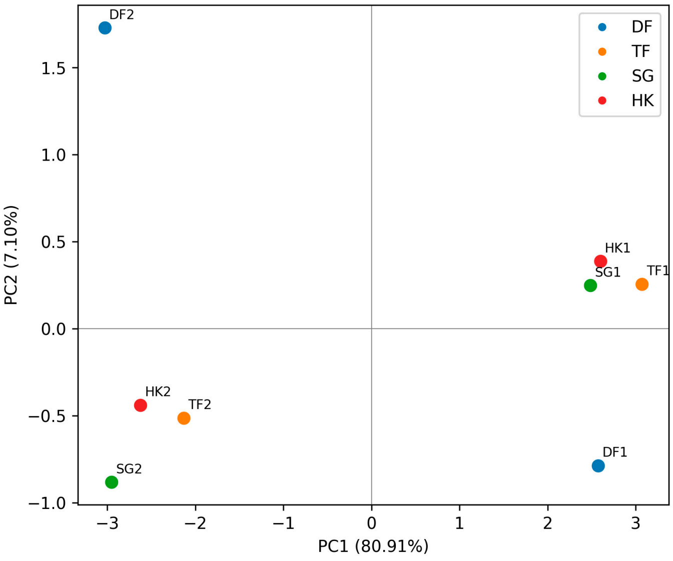

Figure S6 The PCA score plots of the four fermentation production lines, where sample numbered 1 represents Raw Pu-erh and sample numbered 2 represents RpPT

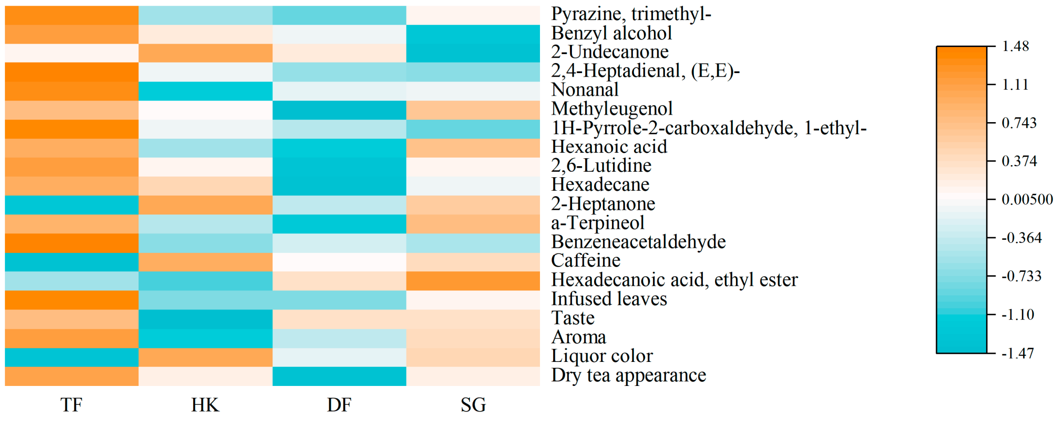

Figure S7 RpPT sensory evaluation score and standardized heatmap of representative metabolites' Z-scores

Table S1

The changes of physicochemical properties during fermentation of RpPT.

Sample	SG		HK		TF		DF	
	**Raw Pu-erh**	**RpPT**	**Raw Pu-erh**	**RpPT**	**Raw Pu-erh**	**RpPT**	**Raw Pu-erh**	**RpPT**
Moisture Content (%)	4.92±0.15	7.54±0.07***	4.82±0.2	6.74±0.07***	4.35±0.2	6.59±0.08***	6.87±0.1	6.49±0.17**
Water Extract(%)	47.26±1.05	32.96±1.28***	46.66±0.24	31.26±0.54***	50.01±0.77	31.18±0.55***	50.86±0.4	31.92±0.42***
Tea Polyphenols(%)	28.5±0.44	15.44±0.23***	29.13±0.28	13.43±0.3***	30.43±0.14	8.33±1.12***	31.62±1.02	11.27±0.11***
Free Amino Acids(%)	3.15±0.02	1.27±0.02***	3±0.04	1.08±0.03***	3.81±0.03	1.11±0.02***	3.42±0.05	1.07±0.02***
Flavonoids(%)	1.08±0.02	1.55±0.02***	1.03±0.03	1.56±0.04***	1.32±0.04	1.55±0.03***	1.14±0.02	1.42±0.03***
Soluble Sugars(%)	3.35±0.05	1.62±0.14***	3.53±0.08	2.03±0.12***	4.7±0.02	3.27±0.15***	4.43±0.03	2.62±0.12***
Theaflavins (TF)(%)	0.24±0	0.1±0.01***	0.21±0.02	0.1±0.01***	0.25±0.01	0.12±0***	0.29±0	0.29±0.01***
Thearubigins (TR)(%)	5.04±0.13	2.73±0.27***	4.85±0.21	1.67±0.11***	4.77±0.07	3.57±0.2***	6.09±0.16	3.27±0.3***
Theabrownins (TB) (%)	2.28±0.05	10.52±0.7***	2.32±0.09	11.63±0.53**	2.24±0.07	9.98±0.37***	2.6±0.09	8.6±0.34***
Catechin(mg/g)	3.69±0.03	1.24±0.12***	4.66±0.03	0.29±0.03***	5.98±0.34	0.76±0.07***	9.49±0.01	1.11±0.13***
Catechin Gallate(mg/g)	0.25±0	0.47±0.05**	0.23±0	0.08±0***	0.47±0.06	0.11±0.14**	0.31±0.01	0.51±0.03***
Epicatechin(mg/g)	8.78±0.08	2.6±0.16***	11.25±0.09	0.28±0.01***	11.88±0.45	1.11±0.1***	13.56±0.07	1.98±0.38***
Epicatechin Gallate(mg/g)	22.19±0.18	1.62±0.15***	22.15±0.07	0.47±0.02***	25.19±1.08	0.83±0.43***	23.22±0.19	2.07±0.83***

Epigallocatechin(mg/g)	28.62±0.41	7.03±1.73***	25.5±0.58	0±0***	7.8±0.5	7.02±0.68	13.79±0.35	2.92±0.82***
Epigallocatechin Gallate(mg/g)	42.87±0.22	2.21±0.14***	42.94±0.12	2.29±0.06***	57.45±4.71	3.1±0.34***	41.6±0.54	2.17±0.09***
Gallic Acid(mg/g)	0.63±0	8.34±0.27***	0.52±0.01	5.26±0.05***	0.59±0.03	7.35±0.17***	0.62±0.01	5.83±0.35***
Gallocatechin(mg/g)	0.83±0.01	1.48±0.08***	1.96±0.02	0±0***	3.35±0.12	3.27±0.03	2.83±0.03	0±0***
Gallocatechin Gallate (mg/g)	0.43±0.01	0.33±0.07	0.59±0	0.06±0.01***	0.4±0.02	0.32±0.1	0.78±0.02	0±0***
Theophylline(mg/g)	0.09±0.02	0.16±0.04	0.1±0	0.46±0.01***	0.2±0.01	0.19±0.02	0.08±0	0.2±0***
Quercetin(mg/g)	0.03±0	0.02±0***	0.05±0	0±0***	0.1±0	0.05±0***	0.07±0	0±0**
Taxifolin (Dihydroquercetin)(mg/g)	0.29±0	0.46±0.08**	0.47±0	0.11±0***	0.52±0	0.64±0.13	0.55±0.01	0.7±0.02***
Caffeine(mg/g)	38.06±2.47	37.4±1.33	32.91±1.04	28.31±2.21*	35.3±1.12	38.13±2.92	32.39±1.3	38.85±0.94**
Rutin(mg/g)	2.83±0.02	1.96±0.09***	1.63±0.81	0.3±0.02***	2.52±0.15	1.21±0.26***	2.05±0.8	1.41±0.26***
Luteolin(mg/g)	0.09±0	0.07±0**	0.1±0	0.02±0***	0.1±0.07	0.1±0***	0.11±0	0.04±0.01
Kaempferol(mg/g)	0.01±0	2.48±0.1***	0.01±0	0±0***	0.01±0	3.55±0.23***	0.01±0	0.02±0**
Myricetin(mg/g)	0.17±0.04	0.43±0.14**	0.2±0.01	0±0***	0.98±0.08	0.15±0.02***	0.17±0.02	0.51±0.09**

Note: Data are presented as mean ± standard deviation (n = 3). *$p < 0.05$, **$p < 0.01$, ***$p < 0.001$.vs. Raw Pu-erh

Table S2

The E-Nose sensing values of four production lines for RpPT

Serial number	Sensor Name	Performance Description	Sample	Response Value
S1	W1C	Sensitive to aromatic components and benzenoids	TF	$0.94±0.01^{d}$
			SG	$0.91±0.02^{c}$
			DF	$0.86±0.03^{b}$
			HK	$0.83±0.02^{a}$
S2	W5S	Highly sensitive to nitrogen oxides	TF	$3.35±0.14^{a}$
			SG	$3.04±0.3^{b}$
			DF	$2.86±0.22^{bc}$
			HK	$2.61±0.11^{c}$
S3	W3C	Sensitive to aromatic components and amines	SG	$0.9±0.02^{a}$
			TF	$0.88±0.01^{a}$
			DF	$0.86±0.02^{b}$
			HK	$0.85±0^{b}$
S4	W6S	Sensitive to hydrides	HK	$1.22±0.01^{a}$
			TF	$1.15±0^{b}$
			SG	$1.17±0.02^{b}$
			TF	$1.15±0^{c}$
S5	W5C	Sensitive to short-chain alkanes and aromatic components	TF	$0.93±0^{a}$
			SG	$0.91±0.01^{a}$
			DF	$0.89±0.02^{b}$
			HK	$0.87±0.01^{c}$
S6	W1S	Sensitive to methyl-containing compounds	HK	$2.27±0.17^{a}$
			DF	$1.98±0.19^{b}$
			SG	$1.6±0.17^{c}$
			TF	$1.41±0.04^{c}$
S7	W1W	Sensitive to sulfur compounds	TF	$8.85±0.46^{a}$
			DF	$7.35±1.52^{ab}$
			HK	$7.1±0.5^{b}$
			SG	$6.58±1.53^{b}$
S8	W2S	Sensitive to alcohols, aldehydes, and ketones	HK	$1.96±0.15^{a}$
			DF	$1.6±0.14^{b}$
			SG	$1.39±0.08^{c}$
			TF	$1.22±0.02^{d}$
S9	W2W	Sensitive to aromatic components and organosulfur compounds	TF	$2.43±0.08^{a}$
			HK	$2.32±0.09^{a}$
			DF	$2.3±0.22^{ab}$
			SG	$2.08±0.25^{c}$
S10	W3S	Sensitive to long-chain alkanes	HK	$1.79±0.01^{a}$
			DF	$1.59±0.05^{b}$
			SG	$1.54±0.05^{c}$

TF	1.43 ± 0.01^{d}
